# Niche conservatism and evolution of climatic tolerance in the Neotropical orchid genera *Sobralia* and *Brasolia* (Orchidaceae)

**DOI:** 10.1038/s41598-022-18218-4

**Published:** 2022-08-17

**Authors:** Marta Kolanowska, Spyros Tsiftsis, Magdalena Dudek, Kamil Konowalik, Przemysław Baranow

**Affiliations:** 1 Department of Geobotany and Plant Ecology, Faculty of Biology and Environmental Protection, University of Lodz, Banacha 12/16, 90-237 Lodz, Poland; 2 Department of Biodiversity Research, Global Change Research Institute AS CR, Bělidla 4a, 603 00 Brno, Czech Republic; 3 Department of Forest and Natural Environment Sciences, International Hellenic University, 1st km Drama, 66132 Microchori, Greece; 4 Department of Plant Taxonomy and Nature Conservation, University of Gdańsk, ul. Wita Stwosza 59, 80-308 Gdańsk, Poland; 5 Department of Plant Biology, Institute of Environmental Biology, Wrocław University of Environmental and Life Sciences, Kożuchowska 5b, 51-631 Wrocław, Poland

**Keywords:** Evolution, Plant sciences, Ecology

## Abstract

*Sobralia* and *Brasolia* form a large complex of Neotropical Orchidaceae. Although the molecular and morphological studies allowed to increase the rate of work on the modern classification of the taxa, they still require the attention as remaining without complete revision. The niche similarity analysis between representatives of *Sobralia* and recently segregated from this taxon—genus *Brasolia* is presented. The ecological tolerance evolution within the group was investigated with molecular clock analysis and phylogeny as the background. The phylogenetic analysis has confirmed the previous results and placed *Brasolia* representatives in a single clade with *Elleanthus* and *Sobralia* core as a separated group. The molecular clock analysis suggests that *Sobralia* and *Brasolia* are relatively young groups that evolved between 8.5 and 8 million years ago. Distribution of suitable niches of studied species is generally congruent with the known geographical ranges of particular taxa. The calculated niche overlap did not indicate any correlation between niche overlap and species phylogenetic relationships and remains low for both intra- and intergeneric relationships. The reconstruction of climatic tolerance evolution indicated that the studied species of *Brasolia* and *Sobralia* are characterized by generally similar ecological tolerance for most of the analyzed variables.

## Introduction

The importance of ecology in speciation has been discussed since Darwin’s^1^ theory on the evolution of species. The divergent selection of ecologically affected characters can lead to the reproductive isolation of populations inhabiting separated niches. Grafen^2^ suggested that niche similarity of closely related species is a result of common ancestry, as well as their shared environmental preferences. The recent advancements in the studies on ecological niches prompted scientists to intensify research on the relationship between fundamental niches and the evolution of various organisms^3–5^. Substantial dispute has emerged in the literature about phylogenetic niche conservatism (PNC), which was defined as the tendency of lineages to retain their ancestral niche-related traits through speciation events^6–8^. Although numerous scientists consider PNC pattern to be common in nature^9^, several case studies indicated that ecological and phylogenetic similarities often are not correlated^10,11^.

Without a doubt, the currently observed diversity of numerous orchids is the result of rapid radiation, which supposedly was the result of their adaptation to various pollinators^12–14^. However, the importance of environmental factors in the diversification of Orchidaceae has been poorly studied^15^.

In this paper, the niche similarity between representatives of *Sobralia* Ruiz & Pav. and recently segregated from this taxon, genus *Brasolia* (Rchb.f.) Baranow, Dudek & Szlach. was evaluated. Moreover, the evolution of ecological tolerances within these orchids was reconstructed. To analyze the impact of preferred ecological niches on the speciation and evolution of *Sobralia* and *Brasolia,* we conducted both phylogenetic study and ecological niche modeling.

The studied group consists of about 200 species with characteristic reed-like stems reaching from over a dozen centimeters to more than 10 m high, often forming dense clumps. The position and structure of the inflorescence are diversified. Along with the other morphological characters and the phylogenetic studies results, it was the argument for proposition of division *Sobralia* into two genera with *Brasolia* as a group with branching inflorescences and *Sobralia* with unbranched, often condensed inflorescences^16,17^. The flowers of *Brasolia* are often somewhat fleshy, persistent, often with contrasting colored fimbriate keels on the labellum, opening simultaneously or successively but always more than one at a time on the inflorescence and with developing floral buds visible. Flowers of *Sobralia* are more delicate, in most cases ephemeral and short-lasting, usually with two keels or ridges in the throat, sometimes with some other projections on the disc. Only species of the section *Racemosae* Brieger have elongated inflorescence with visible internodes. In this section, flowers at various stages of development can be observed at a time. The remaining species have condensed, cone-like inflorescences with imbricating floral bracts and usually one, sometimes two flowers at a time. The floral buds appear after the previous flowers fall off. The group with abbreviated inflorescences is the largest one and it is divided into few sections—the nominal one is represented by often tall, reed-like plants with shortened inflorescences covered by tightly imbricating floral bracts forming a cone-like structure. Their flowers are comparatively large as for the studied group, delicate, thin-textured, usually short-lasting, often one day only. The section *Globosae* Brieger is defined as a group of species with narrow, acuminate leaves, narrow base of the column and the cluster of floral bracts increasing in length as flowers are produced reaching 3–4 cm in length. The section *Intermediae* Brieger groups a complex of species with small flowers and inflorescences. Apart from the sections, there are few species or groups of species without the sectional membership. *Sobralia macrophylla* Rchb.f. and its allies are characterized by the clustered floral bracts hidden between the leaf-like bracts forming a funnel. *Sobralia undatocarinata* C. Schweinf. group is characterized by loose bract clusters and 2–5 relatively durable flowers produced simultaneously. The description of variety within *Sobralia,* requires also a mention of *S. amabilis* (Rchb. F.) L. O. Williams and *S. callosa,* which differ from the members of the nominal section by inconspicuous lip throat and the column structure^18^.

The studied plants grow in various habitats as epiphytic, terrestrial or lithophytic herbs at various elevations in Central and South America^19^. Their usually relatively large flowers are pollinated by bees or hummingbirds^20^.

The previous studies concerning the phylogeny of the group didn’t discuss the relations within the group and ecological aspects at the same time. The studied genera are object of taxonomical studies, however they still need more attention. The first steps to resolve the polyphyly of *Sobralia* have been made, and as a result, *Brasolia* was separated from *Sobralia*^17^. However, as it is known, with more data more accuracy can be achieved, we decided to expand the study with the ecological aspects analyses. The hypothesis was that *Sobralia* as a genus with much wider occurrence than *Brasolia* will also have wider potential ranges. *Brasolia* species, which mostly occur in Andean localities, are not so flexible as *Sobralia*—a genus with its representatives at much wider range of elevation and hence, in much more types of habitats. The same assumption was made for the ecological tolerance evolution analyses.

## Results

### Phylogenetic relationships

**Figure 1:**
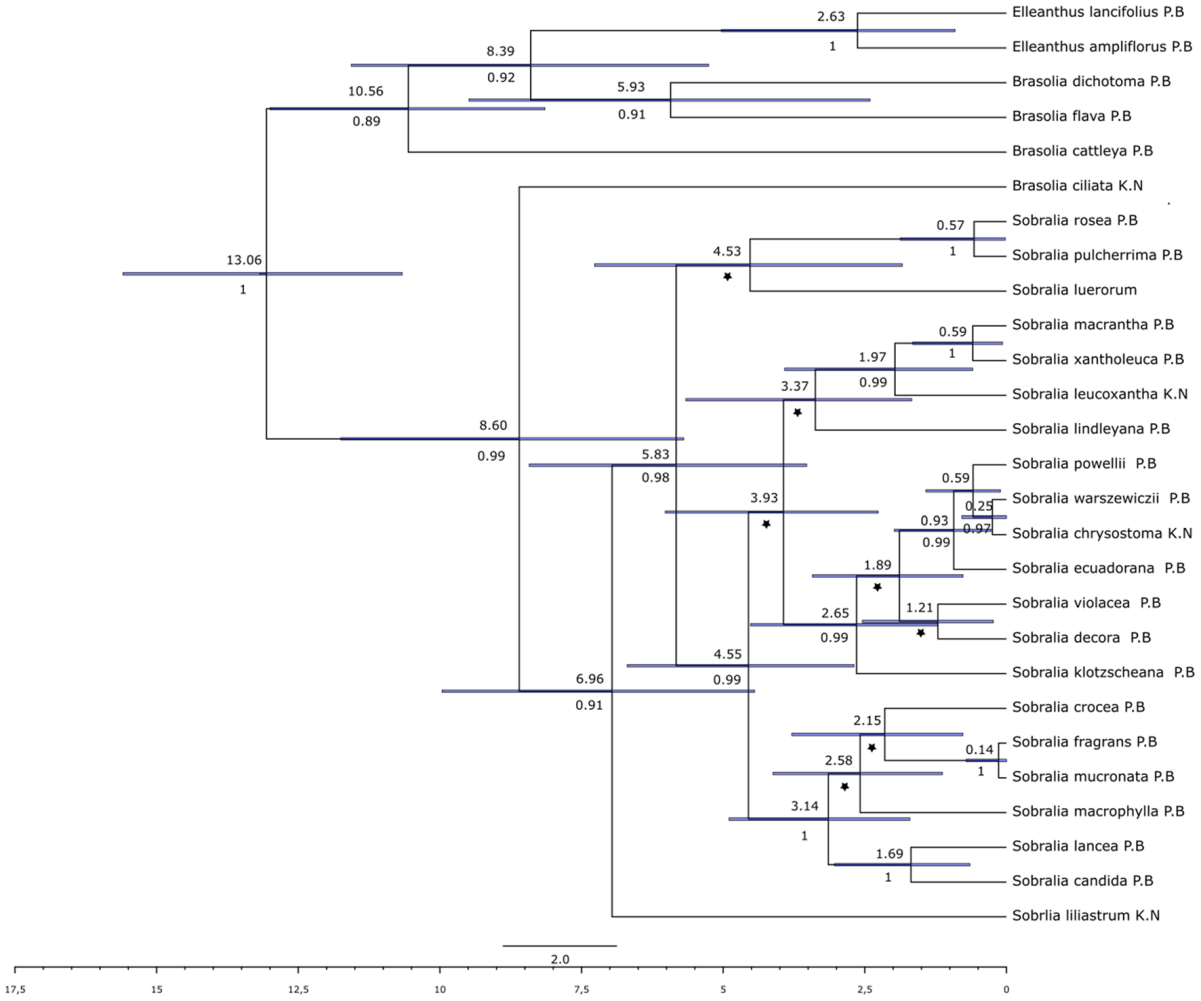
Maximum Clade Credibility tree for *Sobralia* and related genera, Bayesian inference based on nuclear ITS using Beast. The divergence times for each clade were estimated by relaxed molecular clock analysis with Yule model of speciation. The calibration points were designated in the result of phylogenetic analysis of Sobralieae tribe (see supplementary material Fig. S1). The values above nodes present the divergence time, those below the branches are posterior probabilities (PP ≥ 0.9), the values of PP < 0.9 are indicated as a star.

On the phylogenetic tree including the molecular clock analysis results (Fig. 1), the members of the recently appointed *Brasolia* genus [*B. flava* (Baranow & Szlach.) Baranow, Dudek & Szlach., *B. dichotoma* (Ruiz & Pav.) Baranow, Dudek & Szlach. and *B. cattleya* (Rchb. f.) Baranow, Dudek & Szlach.] form a separate group, which seems to be a basal clade for the whole phylogenetic tree. Only *Brasolia ciliata* (C. Presl) Baranow, Dudek & Szlach. is clustered with the members of *Sobralia* but the members of the latter form a sister group for the taxon. If considering the genus *Sobralia*, the section *Racemosae* (*S. liliastrum* Lindl., *S. luerorum* Dodson, *S. pulcherrima* Garay and *S. rosea* Poepp. & Endl.) forms the basal clades for the whole group. The remaining species form three clades, the most distinct of them is a clade of *Sobralia* section *Globosae* Brieger members (*S. lancea* Garay and *S. candida* (Popp. & Endl.) Rchb. f.), section *Intermediae* Brieger (*S. crocea* (Popp. & Endl.) Rchb. f., *S. mucronata* Ames & C. Schweinf. and *S. fragrans* Lindl.) and unclassified *S. macrophylla* Rchb. f.. The remaining two clades are groups of the nominal section representatives.

The molecular clock analysis suggests (Fig. 1), that *Sobralia* and *Brasolia* are relatively young groups that evolved between 8.5 and 8 Mya (Million years ago) together with *Elleanthus* forming a sister group for the clades composed by the two genera. The most intensive diversification of *Sobralia* and *Brasolia* has started about 3Mya. Their ancestor has diversified into two groups between 6.5 and 6 Mya.

### Ecological niche modeling, limiting factors and niche overlap

All models received high AUC scores of 0.955–1.000 (Table S2). Mapped distribution of suitable niches of studied species created based on current climatic conditions is generally congruent with the known geographical ranges of particular taxa (Annex 1–6). Singular localities of few species (*S. crocea*, *S. decora*, *S. fragrans*, *S. liliastrum*, *S. lindleyana*, *S. powellii*, *S. rosea*, *S. sessilis*, *S. valida*) are outside of the potential range as calculated in MaxEnt.

In some cases, the suitable niche distribution overlaps with the known geographical range of these orchids or the calculated potential range is only slightly broader than known distribution of the plants (*B. dichotoma*, *B. speciosa*, *S. crocea*, *S. leucoxantha*, *S. fragrans*, *S. lindleyana*, *S. luteola*, *S. valida*, *S. wisoniana* and *E. lancifolius*). In other species, the calculated distribution of suitable niches is broader than recognized geographical range.

The distribution of most species is limited mainly by the annual mean temperature (bio1, 16 species), and the annual precipitation (bio12, 10 species). The range of four species is restricted by the precipitation in the driest month (bio14) and the equal number of taxa depends on the precipitation in the warmest quarter (bio18) and the precipitation of the coldest quarter (bio19). The occurrence of *Sobralia pulcherrima* and *Brasolia ciliata* is limited by the mean diurnal range (bio2). The temperature seasonality (bio4) is crucial for occurrence of *Brasolia speciosa*, *Sobralia gloriosa,* and *Sobralia lancea.* The presence of *Sobralia macrantha* depends on the precipitation seasonality (bio15).

**Figure 2:**
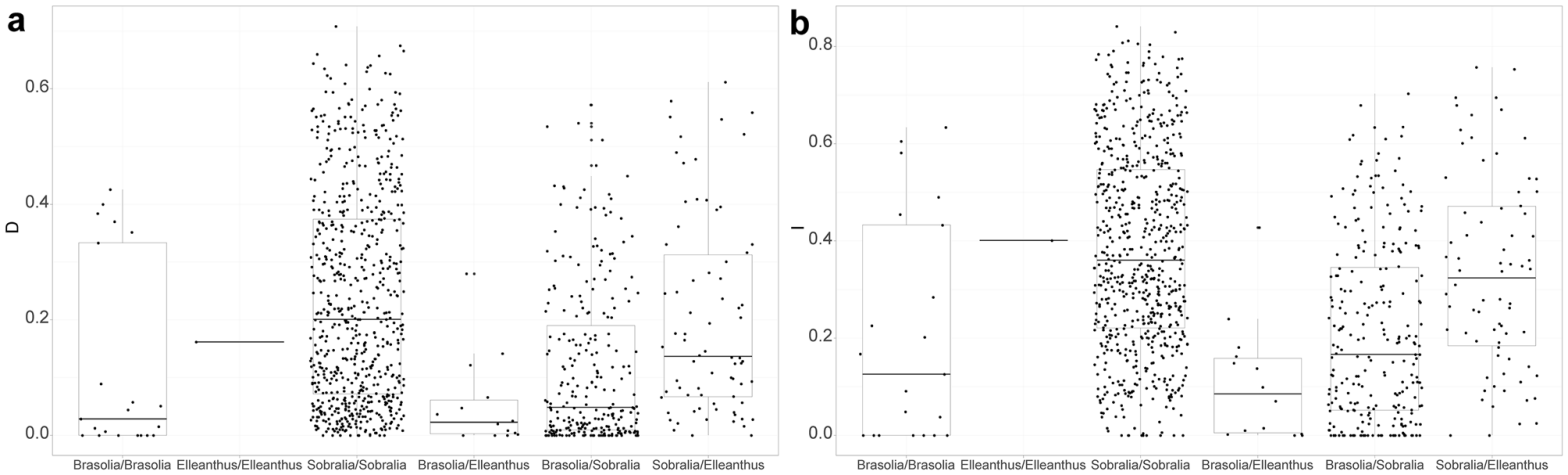
Niche overlap illustrated by (**a**) Schoener’s D^45^ and (**b**) I statistic^46^. Boxplots show the pairwise niche overlap between species belonging to the following groups: within each genus (*Brasolia*, *Elleanthus*, *Sobralia*) and between genera (*Brasolia* and *Elleanthus*, *Brasolia* and *Sobralia*, *Sobralia* and *Elleanthus*). The niche overlap value for each pair of species within a given group is represented by a dot.

The calculated niche overlap (Fig. 2, Tables S2 and S3) did not indicate any correlation between niche overlap and species phylogenetic relationships and remains low for both intra- and intergeneric relationships. This is visible especially within *Brasolia*, whereas in the more species-rich genus *Sobralia* the general overlap is low but there are both species with very low overlap and some that reach values around 0.8 for both D and I indices. Concerning intergeneric relationships, the lowest overlap may be observed between *Brasolia* and *Elleanthus*. Many species belonging to *Brasolia* and *Sobralia* also exhibit a low overlap but this is not true for all pairs of species, as some receive overlap around 0.5 for both D and I indices. Niche overlap between *Sobralia* and *Elleanthus* is generally shifted towards more overlapping niches than between other pairs but still the values are low and their mean oscillates around 0.2 for D statistic and around 0.3 for I statistic.

Within the studied *Brasolia* species, the highest overlap is observed between the pairs *B. cattleya*—*B. ciliata* (I = 0.633, D = 0.426), and *B. ciliata*—*B. flava* (I = 0.581, D = 0.400). The zero value of overlap was calculated for the pairs *B. rupicola*—*B. speciosa* and *B. rupicola*—*B. stenophylla*. In the group *Sobralia Intermediae* + *S. valida,* the highest overlap is observed between the pair *S. valida*—*S. mucronata* (I = 0.726, D = 0.573), while the smallest similarity was detected for the pairs *S. mucronata*—*S. suavolens* (I = 0.298, D = 0.163) and *S. suavolens*—*S. crocea* (I = 0.386, D = 0.168). Within the group *Sobralia Racemosae* the highest overlap is observed between the pair *S. liliastrum*—*S. elisabethae* (I = 0.646, D = 0.532), while the smallest similarity was detected for the pairs *S. granitica*—*S. gloriosa* (I = 0.181, D = 0.047), *S. granitica*—*S. rosea* (I = 0.209, D = 0.0451), and *S. granitica*—*S. pulcherrima* (I = 0.175, D = 0.0327).

### Ancestral climatic suitability and evolution of climatic tolerance

**Figure 3:**
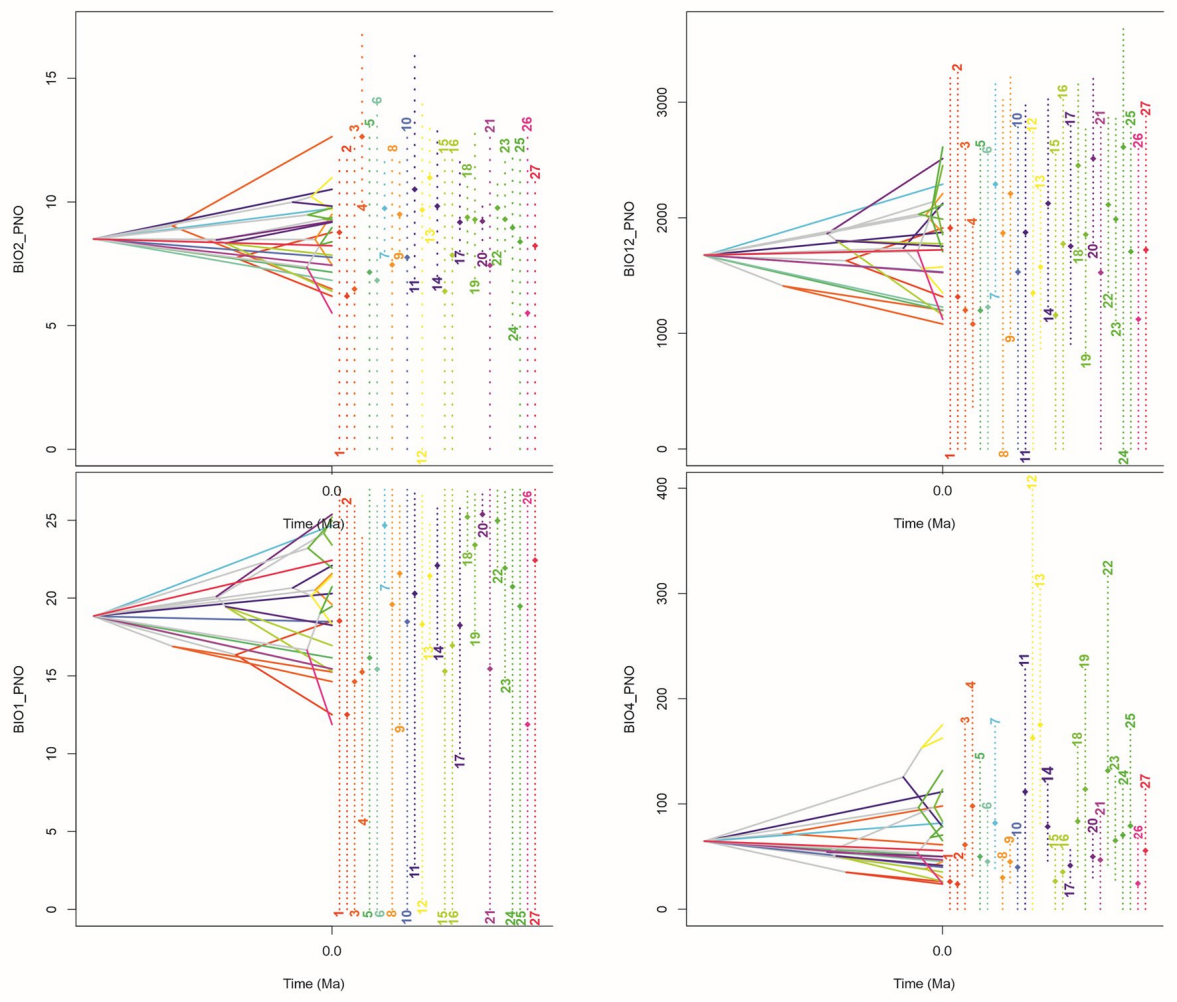
Ecological tolerance evolution for BIO1 (Annual Mean Temperature), BIO2 (Mean Diurnal Range), BIO4 (Temperature Seasonality), BIO12 (Annual Precipitation); 1—*E. lancifolius*, 2—*E. ampliflorus*, 3—*B. flava*, 4—*B. dichotoma*, 5—*B. cattleya*, 6—*B. ciliata*, 7—*S. liliastrum*, 8—*S. pulcherrima*, 9—*S. rosea*, 10—*S. luerorum*, 11—*S. lindleyana*, 12—*S. xantholeuca*, 13—*S. macrantha*, 14—*S. leucoxantha*, 15—*S. lancea*, 16—*S. candida,* 17—*S. crocea*, 18—*S. fragrans*, 19—*S. mucronata*, 20—*S. macrophylla*, 21—*S. klotzscheana*, 22—*S. decora*, 23—*S. violacea*, 24—*S. chrysostoma*, 25—*S. warszewiczii*, 26—*S. ecuadorana*, 27—*S. powellii.* The total time scale = 42.87 Ka.

**Figure 4:**
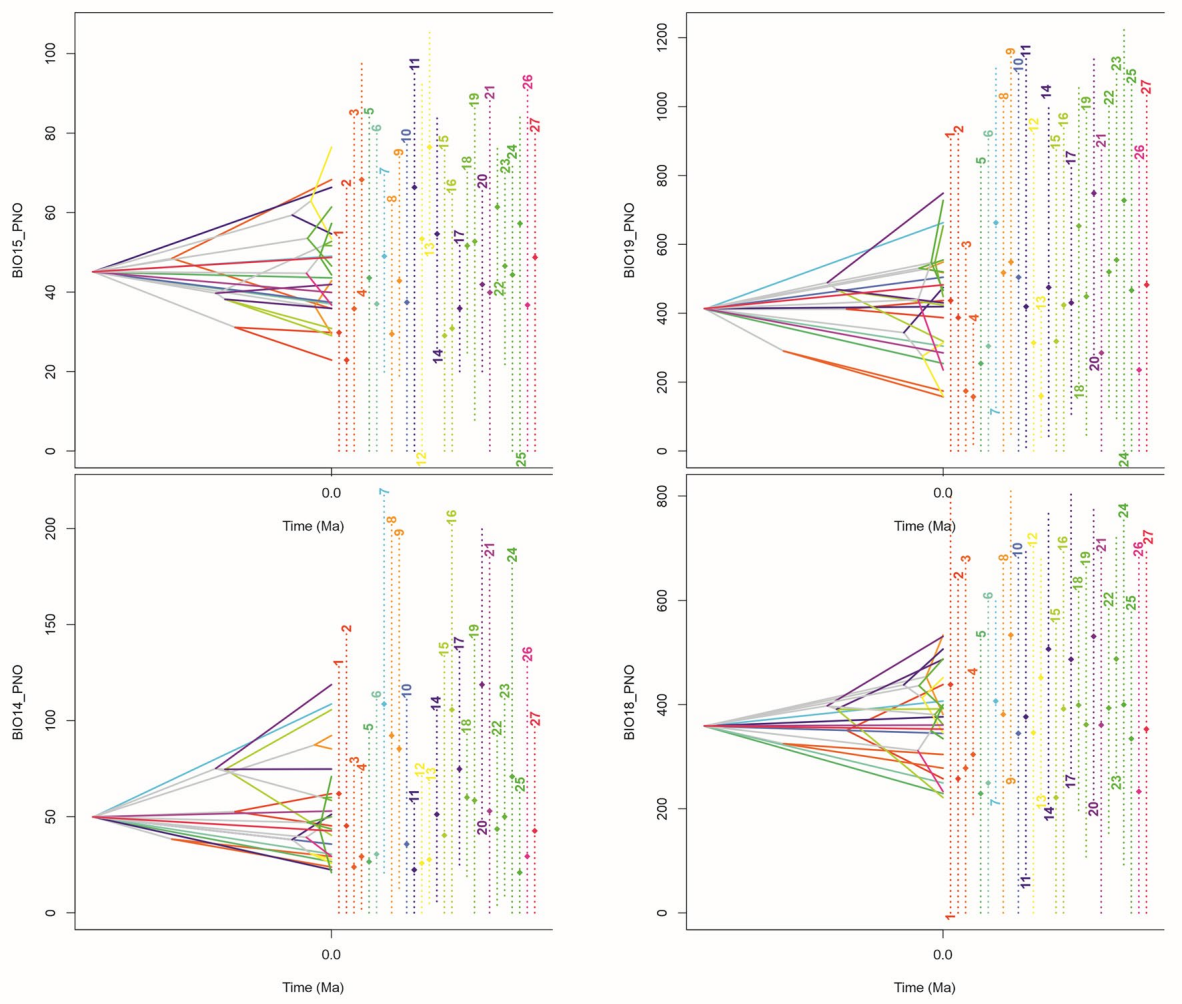
Ecological tolerance evolution for, BIO14 (Precipitation of Driest Month), BIO15 (Precipitation Seasonality), BIO16 (Precipitation of Wettest Quarter), BIO19 (Precipitation of Coldest Quarter); 1—*E. lancifolius*, 2—*E. ampliflorus*, 3—*B. flava*, 4—*B. dichotoma*, 5—*B. cattleya*, 6—*B. ciliata*, 7—*S. liliastrum*, 8—*S. pulcherrima*, 9—*S. rosea*, 10—*S. luerorum*, 11—*S. lindleyana*, 12—*S. xantholeuca*, 13—*S. macrantha*, 14—*S. leucoxantha*, 15—*S. lancea*, 16—*S. candida,* 17—*S. crocea*, 18—*S. fragrans*, 19—*S. mucronata*, 20—*S. macrophylla*, 21—*S. klotzscheana*, 22—*S. decora*, 23—*S. violacea*, 24—*S. chrysostoma*, 25—*S. warszewiczii*, 26—*S. ecuadorana*, 27—*S. powellii.* The total time scale = 42.87 Ka.

The reconstruction of climatic tolerance evolution indicated that the studied species of *Brasolia* and *Sobralia* are characterized by generally similar ecological tolerance for most of the analyzed variables and that their ecological divergence began c. 42.87 Kya ago. However, we did not find any correlation between the phylogeny and the ecological tolerance—in most cases, the closely related taxa are not characterized by more similar niches than more distinct species. However, both included *Elleanthus* species seem to have similar tolerance which may be different than in most of *Brasolia* and *Sobralia* species (bio4). It may suggest that the extended sampling could bring the new conclusions concerning the ecological tolerance—phylogeny relation. Noteworthy, most species share at least a part of climatic niches and we did not identify any species which adapted to completely separated values of analyzed variables (Figs. 3, 4).

The first conclusion is that some species have a narrow tolerance considering the analyzed variables. The examples are *Sobralia macrantha* and *S. crocea*. On the contrary, some species are characterized by wide tolerance spectrum. *Sobralia luerorum*, *S. klotscheana*, *S. warszewiczii*, *S. ecuadorana* and *S. powellii* are the examples of the case.

If considering the specific ecological factors, it was noticed that some of them are more limited for the studied species than the others. For temperature seasonality (bio4), almost all the studied species indicate the narrow ecological tolerance. On the other hand, considering annual precipitation (bio12), broad spectrum of tolerance for almost all taxa is observed.

It is worthy to emphasize the topology of the diversification of the studied group in order to the time scale and the phylogenetic relations.

## Discussion

Our study indicated strong niche conservatism in *Sobralia* and its relatives. The representatives of various phylogenetic groups are limited by similar climatic factors and apparently their evolution was not directly related to the adaptation to various climatic variables. According to our analyses, the most closely related species do not share the most similar niches, whereas the general ecological tolerance in *Sobralia* and *Brasolia* is relatively narrow considering the tested environmental variables.

Out of the possible radiation triggers pointed by Dodson^21^, the most probable reason of diversification of *Sobralia* and its relatives was the adaptation to various pollinators. Flowers of *Sobralia* and *Brasolia* were reported to be pollinated by a variety of large solitary bees, especially by euglossine bees^22^. However, in some species (e.g. *S. amabilis* and *S. callosa*) pollen is transferred by hummingbirds^18^. The previously published data suggested that when separated by putative pollinator, different groups of Sobralieae showed affinities to the elevational preference of pollinators^23^.

The phylogeny based on the set of taxa used in the analyses (Fig. 1) confirmed the previous phylogenetic study devoted to the discussed taxa relationships^17,24^.

It is worthy to mention, that the molecular clock created for Sobralieae, indicated that the highest rate of diversification occurred in this group recently, between 2 Mya (Million years ago) and the present time. If compared with the phylogeny of euglossine bees^25^, the speciation within *Sobralia* was proceeded by the intensive radiation of the Euglossini. The diversification of insects began in the end of the Miocene and reached the highest rate in the mid-Pliocene, between ca. 5.5 and 2 Mya^25^. In our opinion these two events could be correlated and the diversification of Sobralieae was probably triggered by the increase of pollinators’ diversity. However, to confirm such hypothesis a broader set of observations on *Brasolia* and *Sobralia* pollinators would be required.

In the case of the studied orchid group, epiphytism is not the widespread growth system and the majority of its species grows on the ground, hereby the adaptation to various microhabitats in trees^21^ can be rejected as a radiation trigger. Moreover, Neubig^23^ did not find any one-to-one mimicry related to the food deception within *Sobralia,* so this character should not be considered as related with rapid diversification of the studied orchids.

The associations of *Sobralia* and its relatives with mycorrhizal fungi have not been explored so far. However, considering the variety of environments where the studied orchids occur, including terrestrial and epiphytic habitat, as well as different types of soils, we suppose that the specialization of mycorrhizal partner did not prompt speciation events.

*Sobralia* and its relatives can occur in both lowlands and mountain habitats. However, representatives of this group are most diverse in the montane regions of Neotropics. We believe that the orogeny of cordilleras of Central America and the Andes prompted radiation of pollinators and this event was directly correlated with evolution of pollinator specificity and diversification of *Sobralia*. For example, the intensive uplift of the Eastern Cordillera of the Colombian Andes occurred relatively recently^26^. By 4 Mya, this mountain range reached no more than 40% of its current elevation^26^. The surface uplift of 1400 and 1700 m in the northern Altiplano occurred between 18.7 and 5 Mya^27^. Moreover, several contractional deformations in Central America were dated as quite young^28^. For example, the age of Fila Costeña and Cordillera de Talamanca was estimated to 15–7 Mya and the age of Cordillera Central to 11–7.3 Mya^27^. Undoubtedly, considering the current distribution of some species, e.g. *S. rosea*, the Andes could serve as a North–South corridor for migration of some orchids.

According to our analyses, the studied group is relatively young and its highest rate of speciation began *ca.* 3 Mya. However, the intensive diversification of ecological niche tolerance in *Sobralia* and *Brasolia* started around 50 Kya. This recent evolution of preferred climatic niches was also indicated in some previous study on Orchidaceae^29^. Apparently, the divergence of ecological niche tolerance after the great species diversification is a repeatable phenomenon in tropical and subtropical orchids, whose evolution was driven mainly by adaptation to various pollinators. Since climate adaptation has significant consequences in the evolution and ecology of all living organisms, it would be interesting to evaluate the PNC in *Sobralia* and *Brasolia* pollen vectors and compare it with the diversification of climatic niches of orchids. Arnal et al.^30^ proved that in host‐specialized phytophagous insects, host relationships not always explain niche preferences of insects. So far, no study on the overlap of niches of tropical orchids and their pollinators was conducted and the correlation of the evolution of their ecological tolerances remains unexplored. Unfortunately, this assessment is currently not possible due to the scarce information about species pollinating studied plants.

While all models of suitable niche distribution created for studied orchids received high scores of reliability tests, the potential ranges of some species are clearly broader than the registered geographical distribution of these taxa. Predictions of suitable climatic niches outside of environmental ranges are not uncommon in species which occurrence is related with numerous ecological constrains^31^ and which dispersal is limited by various geographical and biotic barriers^32,33^. Detailed information about availability of specific mycorrhizal fungi and pollinators would have to be used to receive more precise models of potential distribution of the species studied. Unfortunately, the ecology of *Sobralia* and its relatives is so far poorly recognized and the information about its pollen vectors and symbiotic organisms is extremely limited. Here we evaluated exclusively the evolution of climatic niche of *Sobralia* and its relatives, not their realized niches which are also defined by the biotic interactions^34,35^.

## Taxonomic note

***Brasolia rupicola*** (Kraenzl.) Baranow *comb. nov*. **≡ Sobralia rupicola** Kraenzl., Repert. Spec. Nov. Regni Veg. 6: 21. 1908.—TYPE: BOLIVIA. Santa Cruz. Cerro Amboro, Alt. 1400 m. *Herzog 311* (B n.v.).

Notes. Although its morphology allows to classify it within *Brasolia* without doubt, the transfer was missed in the paper elevating *Brasolia* as a separate genus^17^. Thus, we propose the transfer here to keep all the species of the group within one genus.

## Materials and methods

### List of localities

A total of 35 *Sobralia* and 7 *Brasolia* species were included in the ecological niche modelling analyses (Table S1). To evaluate the ancestral climatic tolerance of the studied group, which is based on outcomes of phylogenetic research also information about distribution of two related species, *Elleanthus ampliflorus* Schltr. and *E. lancifolius* C. Presl, were gathered.

The occurrence data were obtained from the information recorded with the specimens deposited in the herbaria AMES (Orchid Herbarium of Oakes Ames), B (Botanischer Garten und Botanisches Museum Berlin-Dahlem, Zentraleinrichtung der Freien Universität Berlin), BM (Natural History Museum, London), COL (Universidad Nacional de Colombia), COAH (Instituto Amazónico de Investigaciones Científicas SINCHI), CUZ (Universidad Nacional San Antonio Abad del Cusco), F (Field Museum of Natural History), K (Royal Botanic Gardens), MA (Real Jardín Botánico), MO (Missouri Botanical Garden), NY (New York Botanical Garden), P (Muséum National d'Histoire Naturelle), U (Naturalis), US (Smithsonian Institution), Z (Universität Zürich), as well as from literature sources and original protologues.

The georeferencing process followed Hijmans et al.^36^. The geographic coordinates provided on the herbarium sheet labels were verified. If there were no geographic coordinates on the herbarium label, we used the description of the locality where the plant was collected and assigned coordinates as precisely as possible to this location. The Google Earth (ver. 6.1.0.5001, Google Inc.) application was used to validate all the information gathered. A total of 786 localities that could be precisely located were used in the ecological niche modeling (ENM) (5–46 localities per species; supplementary material Table S1).

### Ecological niche modelling and niche similarity

The ecological niche modelling was done using the maximum entropy method in MaxEnt version 3.4.3^37–39^ based on presence-only observations of studied species. The duplicate presence records (records within the same grid cell) were removed using MaxEnt.

For the modelling, bioclimatic variables in 2.5 arc-minutes of interpolated climate surface were used. This approach was justified considering the precision of georeferenced records derived from information provided on herbarium material labels. Because some previous studies^40^ indicated that usage of a restricted area in ENM analysis is more reliable than calculating habitat suitability on the global scale, the area of the analysis was restricted to 60°S–35.75°N—126.916°W–31.8749°W.

In this study, as in many other ecological studies, the most widespread source of climatic data was used. WorldClim^41^ is commonly applied to produce species distribution models (> 15,000 citations). Of 19 bioclimatic variables (“bioclims”, Table 1) available in WorldClim (version 2.10^42^ some were removed because they were significantly correlated with one another (above 0.9) as evaluated by Pearsons’ correlation coefficient computed using SDMtoolbox 2.3 for ArcGIS^43^. While the strategy of excluding highly intercorrelated variables has little influence on models derived from MaxEnt according to Feng et al.^44^, because of the reduction of multi-collinearity the following variables were excluded from further analyses: bio3, bio5, bio6, bio7, bio8, bio9, bio10, bio11, bio13, bio17 and bio18.

**Table 1:** Codes of climatic variables developed by Hijmans et al.^41^.

Code	Description
bio1	Annual mean temperature
bio2	Mean diurnal range = Mean of monthly (max temp − min temp)
bio3	Isothermality (bio2/bio7) * 100
bio4	Temperature seasonality (standard deviation * 100)
bio5	Max temperature of warmest month
bio6	Min temperature of coldest month
bio7	Temperature annual range (bio5–bio6)
bio8	Mean temperature of wettest quarter
bio9	Mean temperature of driest quarter
bio10	Mean temperature of warmest quarter
bio11	Mean temperature of coldest quarter
bio12	Annual precipitation
bio13	Precipitation of wettest month
bio14	Precipitation of driest month
bio15	Precipitation seasonality (coefficient of variation)
bio16	Precipitation of wettest quarter
bio17	Precipitation of driest quarter
bio18	Precipitation of warmest quarter
bio19	Precipitation of coldest quarter

In all analyses, the maximum number of iterations was set to 10,000 and convergence threshold to 0.00001. The neutral (= 1) regularization multipler value and auto features were used. All samples were added to the background. The “random seed” option which provided a random test partition and background subset for each run was applied. The run was performed as a bootstrap, which is highly recommended for small-sample analyses, with 1000 replicates, and the output was set to logistic. The evaluation of the created models was made using the most common metric—the area under the curve (AUC)^45–47^. All operations on GIS data were carried out on ArcGis 10.6 (Esri, Redlands, CA, USA) and R^48^.

The quantification of niche overlap between each pair of generated ENMs was done using the ENMTools package for R^48,49^ utilizing two statistics—Schoener’s D^50^ and the I statistic^51^. Calculations were done according to the method proposed by Broennimann et al.^52^, which compares the similarity between two species in an environmental space that is represented by the first and the second axis of the principal component analysis (PCA) computed on bioclimatic variables.

To reconstruct ancestral ecological tolerances and predicted niche occupancy profiles (PNOs), the Phyloclim package was used^53^. This approach implements the methodology originally developed by Evans et al.^54^. PNO profile takes into account species probability of occurrence derived from ecological niche modeling and compiles a response to a particular environmental variable for each species. Ancestral ecological tolerances were computed from the phylogenetic tree and PNO using nonparametric approach and ancestral character estimation^54,55^.

### Phylogenetic analysis

The DNA sequences of ITS region used for molecular analyses were taken from Baranow et al.^17^ and Neubig et al.^24^. They were aligned by Seaview^56^ using MUSCLE algorithm^57^. The complete matrix consisted of 27 taxa representing 21 species of *Sobralia*, 4 species of *Brasolia* and 2 species of *Elleanthus* C. Presl. Data matrix is available from the corresponding author upon request. Gaps were treated as missing data. The molecular substitution model was calculated using MrModeltest 2.2^58^. In both cases, hLRTs and AIC as the best fitting were selected General Time Reversible with gamma distribution and invariable sites (GTR + G + I). For reconstruction of phylogeny we were using MrBayes 3.1.2^59^. The phylogenetic trees were constructed by applying the Bayesian inference using Markov chain Monte Carlo simulations. Four chains were run for 10,000,000 generations of two independent and simultaneous runs. The trees were checked for stability, until the average standard deviation of split ranges was smaller than 0.01. The first 25% trees were discarded as the burn-in. The remaining trees were used to construct a maximum clade credibility tree by using TreeAnnotator v1. 8.1^60^.

To estimate divergence times for representatives of *Sobralia* and *Brasolia*, molecular clock analysis was performed using BEAST 1.8.3^60^. First, based on the nuclear ITS sequences available in GenBank and Baranow et al.^17^, a matrix was prepared for all representatives of Sobralieae Pfitzer with Neottieae Lindl. as an outgroup. The calibration point was chosen according to Givnish et al.^61^ as the time of the last common ancestor for all Epidendroideae (48.05 Mya years ago). Divergence times for representatives of Sobralieae were estimated with a log normal relaxed molecular clock using the Yule model of speciation. Two independent runs, each with 20,000,000 generations of Markov chain Monte Carlo (MCMC) were sampled every 1000 generations. The MCMC output files were probed by Tracer v1.6. The consensus tree files from two runs were obtained with LogCombiner v1.8.1 with a burn-in of 25% of the trees, then the final maximum clade credibility tree was summarized by TreeAnnotator v1.8.1^60^. The obtained results (supplementary material Fig. S1) served us as source of the calibration points to determine the exact divergence times of representatives of *Sobralia* and *Brasolia*. The time of the last common ancestor of Sobralieae tribe was determined to 12.96 Mya years ago (10.60 as 95% HPD lower and 16.50 as 95% HPD upper). The two additional points indicated the time of diversification of *Sobralia* (8.70 Mya) and others member of the tribe (10.98 Mya). We performed the analysis with a log normal relaxed molecular clock using the Yule model of speciation. Two independent runs, each with 10,000,000 generations of Markov chain Monte Carlo (MCMC) were sampled every 1000 generations. The MCMC output files were probed by Tracer v1.6. The consensus tree files from two runs were obtained with LogCombiner v1.8.1 with a burn-in of 25% of the trees, then the final maximum clade credibility tree was summarized by TreeAnnotator v1.8.1^60^.

## Supplementary Information

Supplementary Information 1.

Supplementary Information 2.

Supplementary Information 3.

Supplementary Information 4.

Supplementary Information 5.

Supplementary Information 6.

Supplementary Information 7.

Supplementary Information 8.

## Data Availability

The herbarium specimens list used as the samples for the localities dataset is attached as a supplementary material. All the DNA sequences used for phylogenetic study were taken from previously published results and are available as the cited authors indicated in their works. The alignments used for the presented analysis are available from the corresponding author upon request.
