# Supplementary figures and images for: Niche conservatism and evolution of climatic tolerance in the Neotropical orchid genera Sobralia and Brasolia (Orchidaceae)
Source: Sci Rep. 2022 Aug 17;12:13936. doi: 10.1038/s41598-022-18218-4 (PMC9385687; doi:10.1038/s41598-022-18218-4)

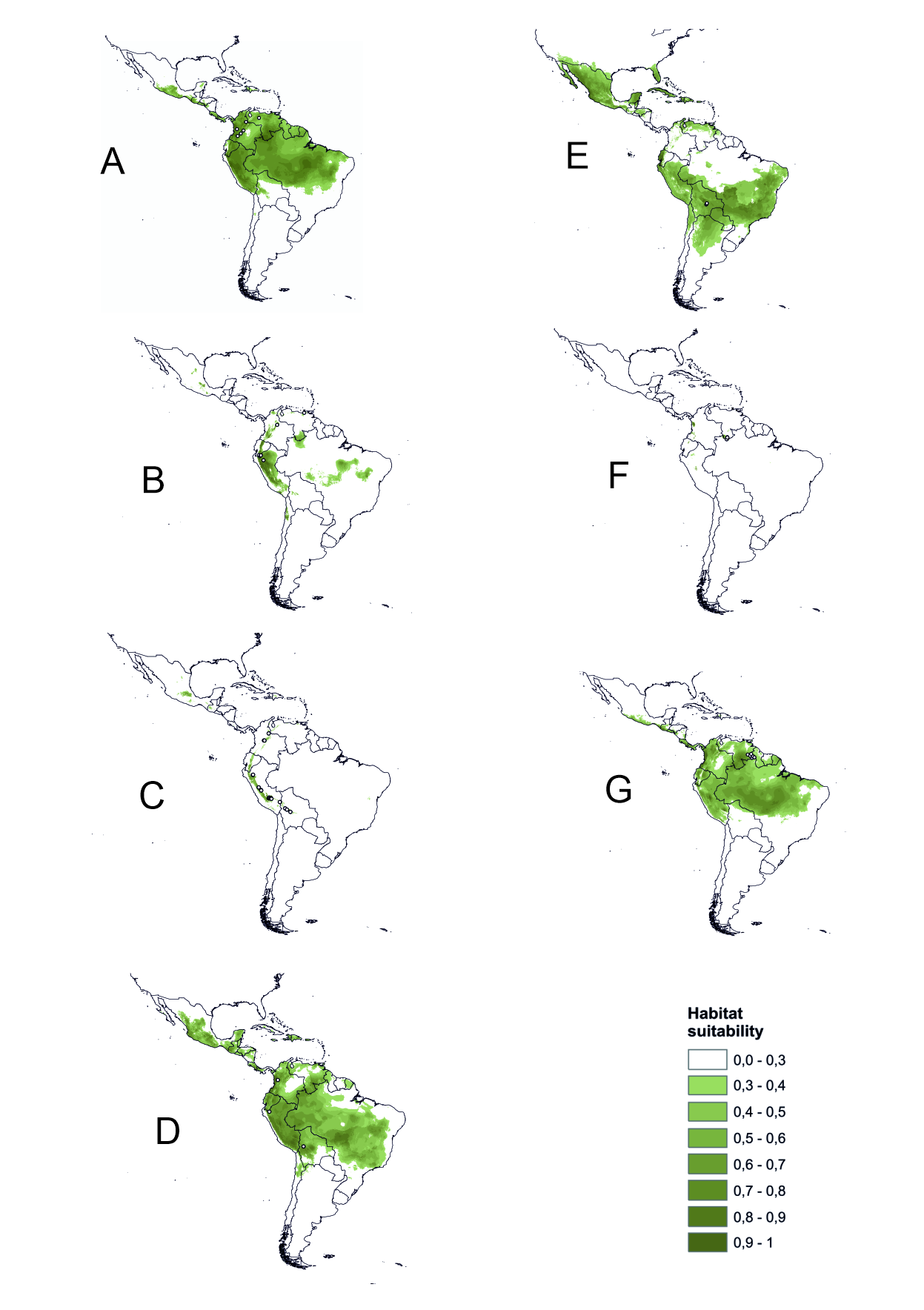

Supplement: Supplementary file 1 — Supplementary Information 1. [file 41598_2022_18218_MOESM1_ESM.tif]

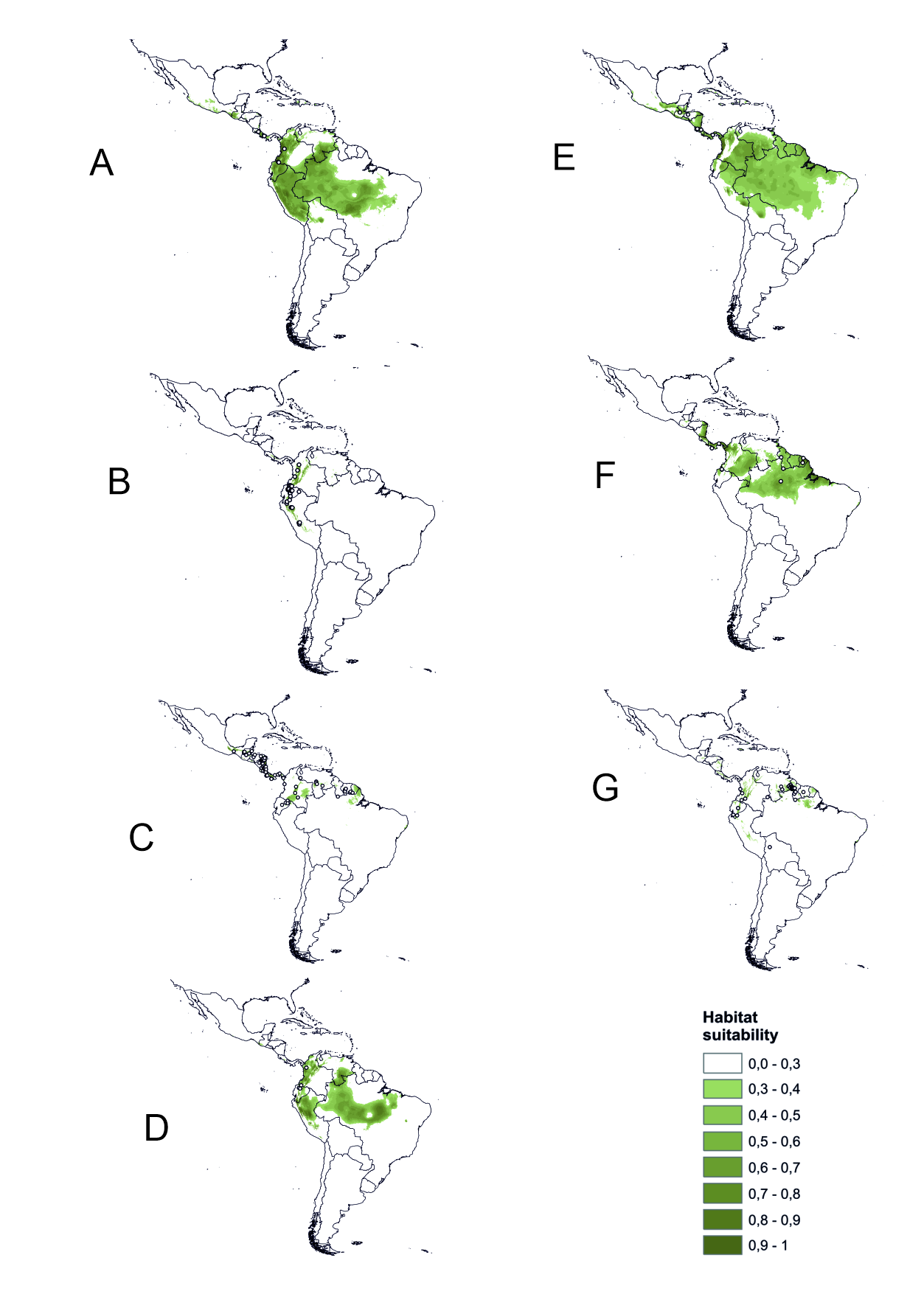

Supplement: Supplementary file 2 — Supplementary Information 2. [file 41598_2022_18218_MOESM2_ESM.tif]

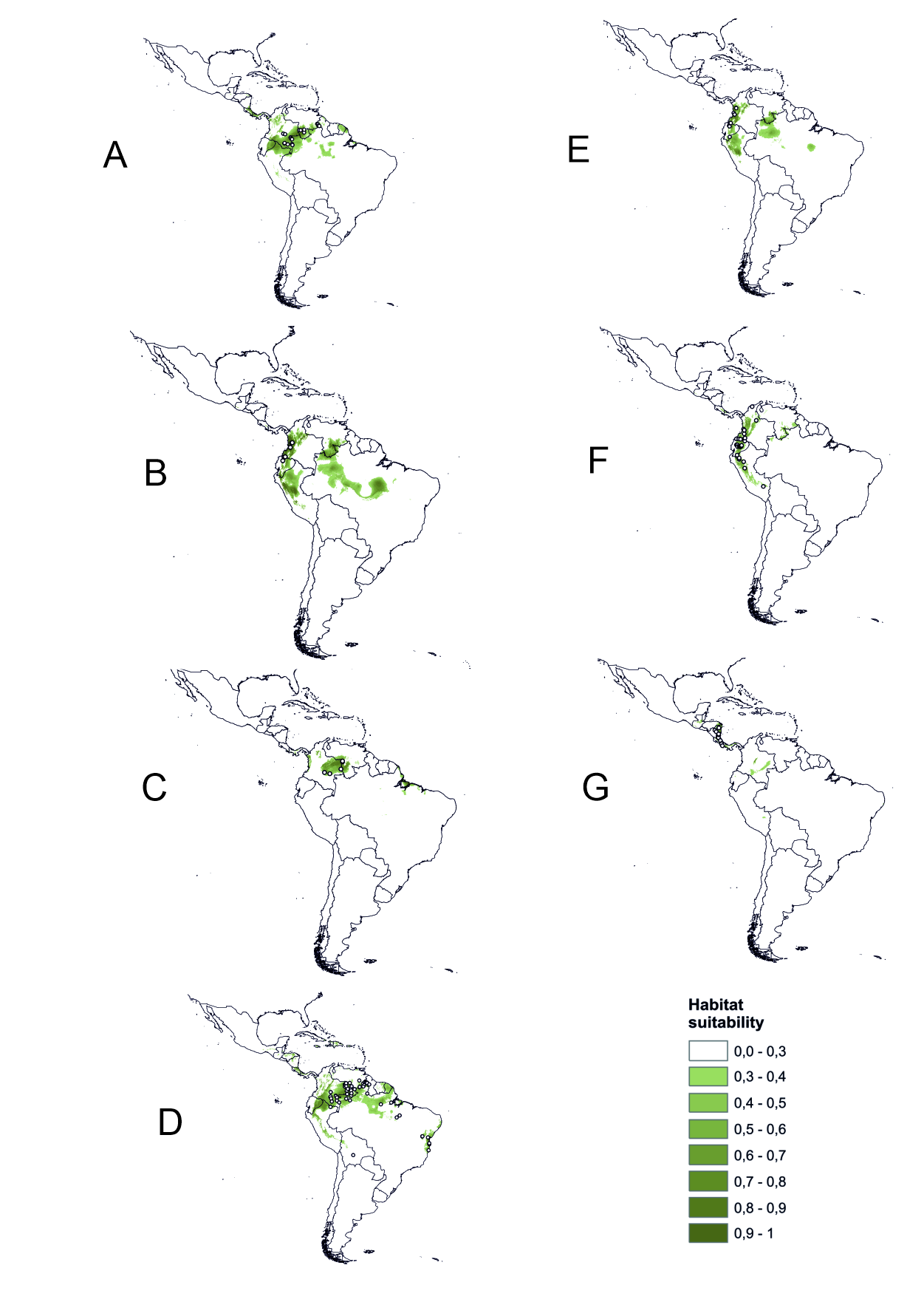

Supplement: Supplementary file 3 — Supplementary Information 3. [file 41598_2022_18218_MOESM3_ESM.tif]

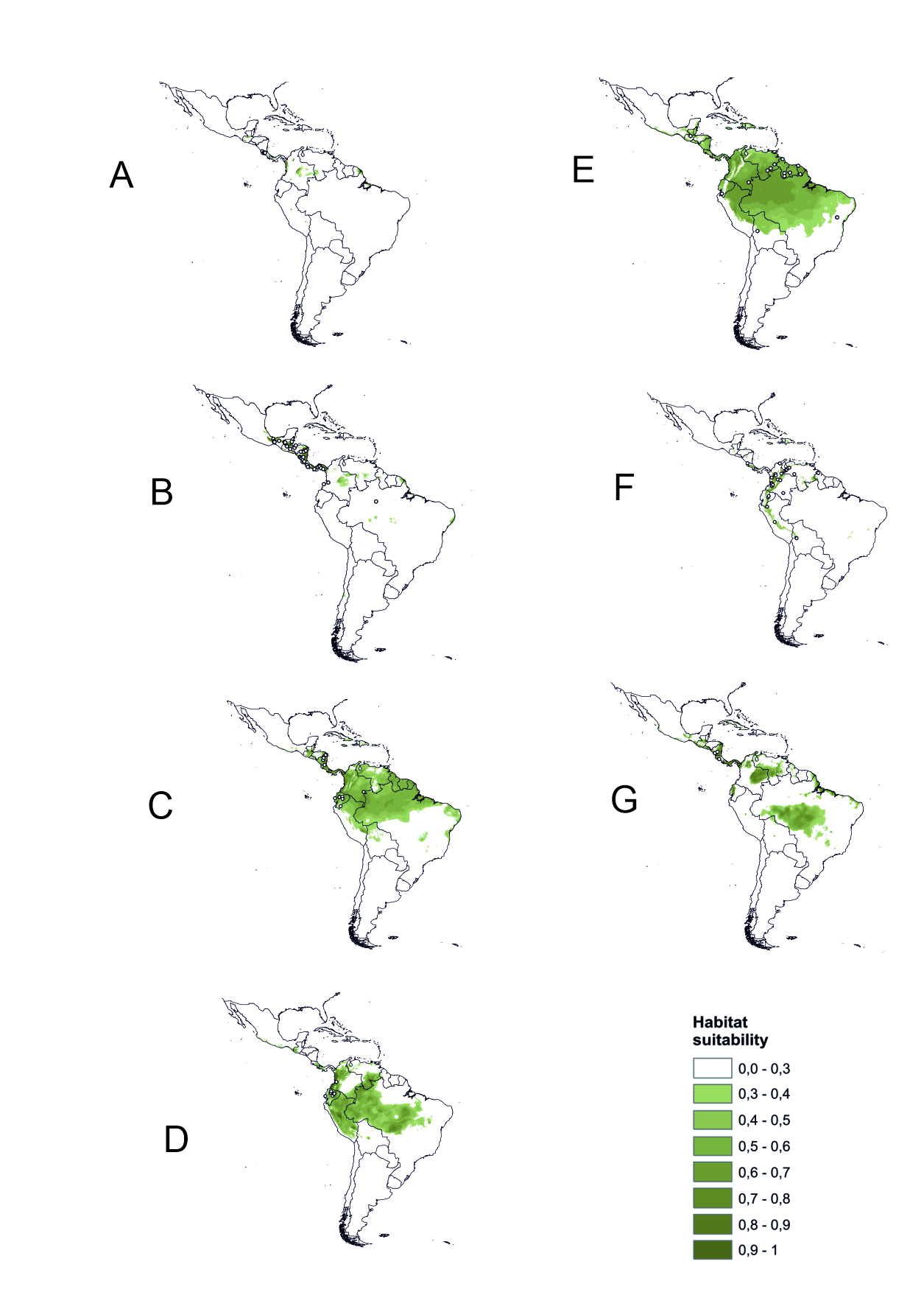

Supplement: Supplementary file 4 — Supplementary Information 4. [file 41598_2022_18218_MOESM4_ESM.tif]

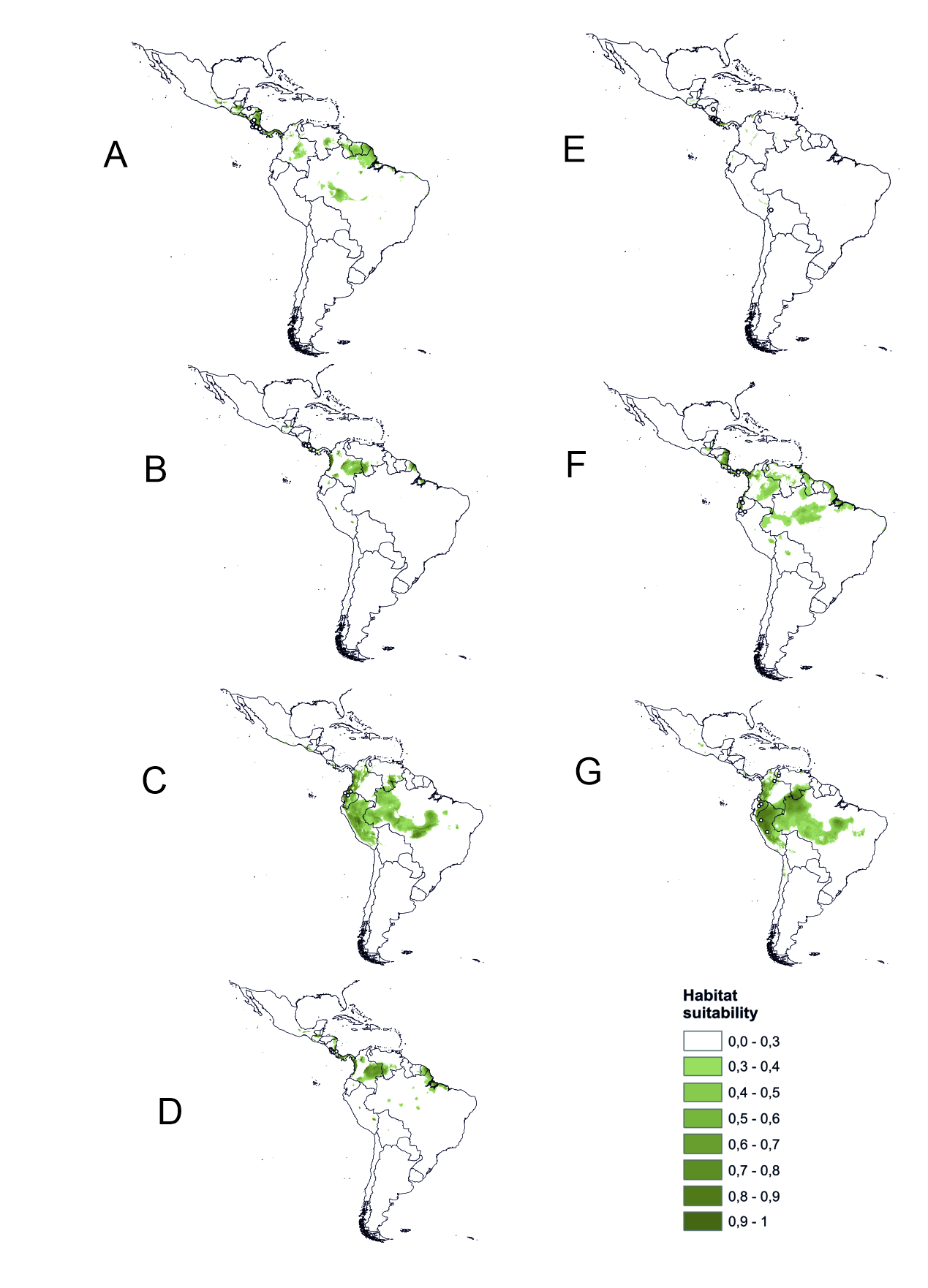

Supplement: Supplementary file 5 — Supplementary Information 5. [file 41598_2022_18218_MOESM5_ESM.tif]

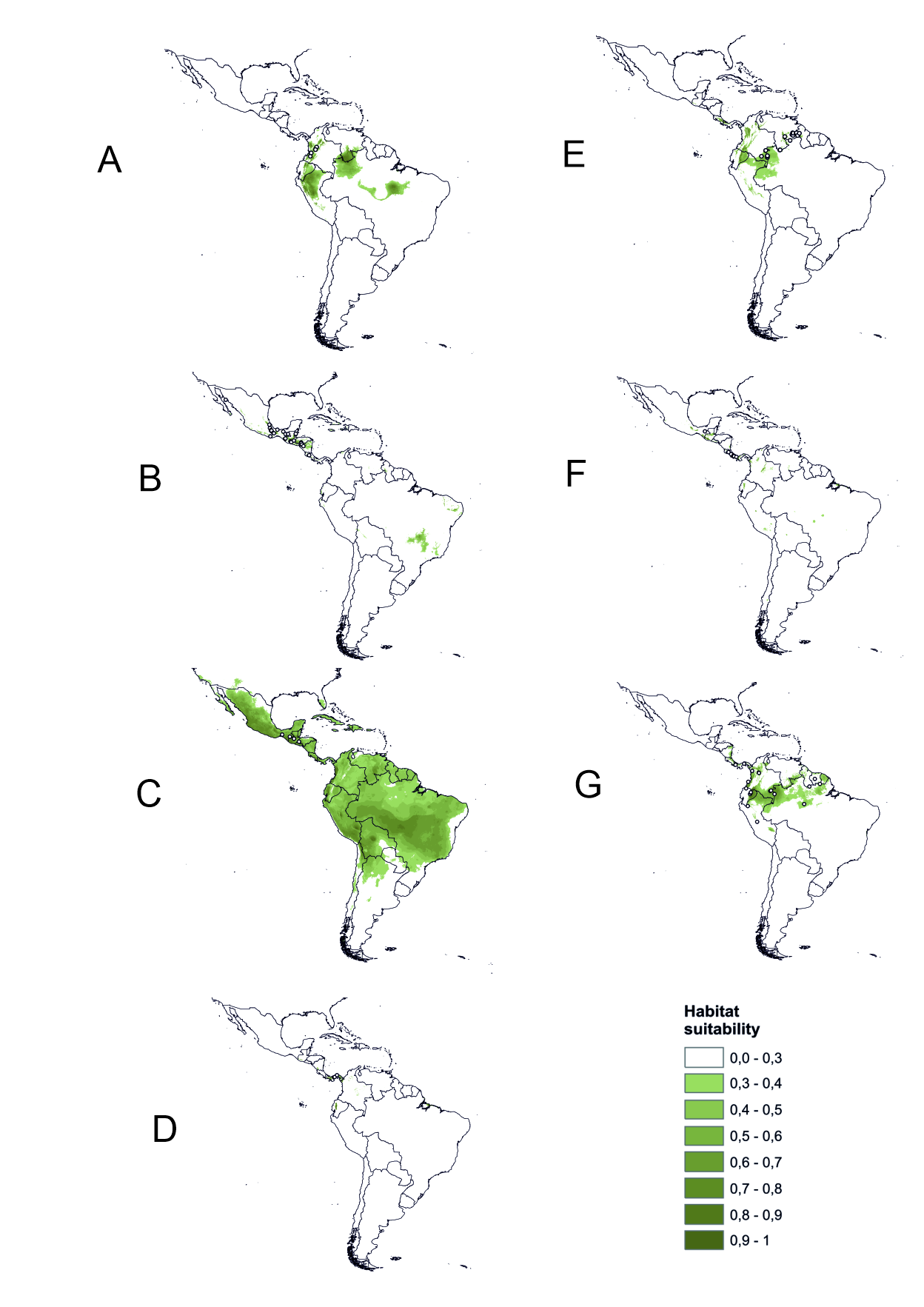

Supplement: Supplementary file 6 — Supplementary Information 6. [file 41598_2022_18218_MOESM6_ESM.tif]
