# Supplementary material for: Niche conservatism and evolution of climatic tolerance in the Neotropical orchid genera Sobralia and Brasolia (Orchidaceae)
Source: Sci Rep. 2022 Aug 17;12:13936. doi: 10.1038/s41598-022-18218-4 (PMC9385687; doi:10.1038/s41598-022-18218-4)
Supplement: Supplementary file 7 — Supplementary Information 7. [file 41598_2022_18218_MOESM7_ESM.pdf]

Niche conservatism and evolution of climatic tolerance in the Neotropical orchid genera Sobralia and Brasolia (Orchidaceae)Marta Kolanowska, Spyros Tsiftsis, Magdalena Dudek, Kamil Konowalik, Przemysław Baranow4

Supplmenetary material Fig. S1.

Phylogenetic relationships of Sobralieae representatives produced by Bayesian inference based on nuclear ITS using Beast. The Maximum Clade Credibility tree was summarized with divergence times. The divergence times for each clade were estimated by relaxed molecular clock analysis with Yule model of speciation. The calibration points were chosen as Givinish *et al*. [56]. The values above nodes present the time of divergence, and those below the branches are posterior probabilities (PP≥0.9), the values of PP <0.9 are indicated as a star.

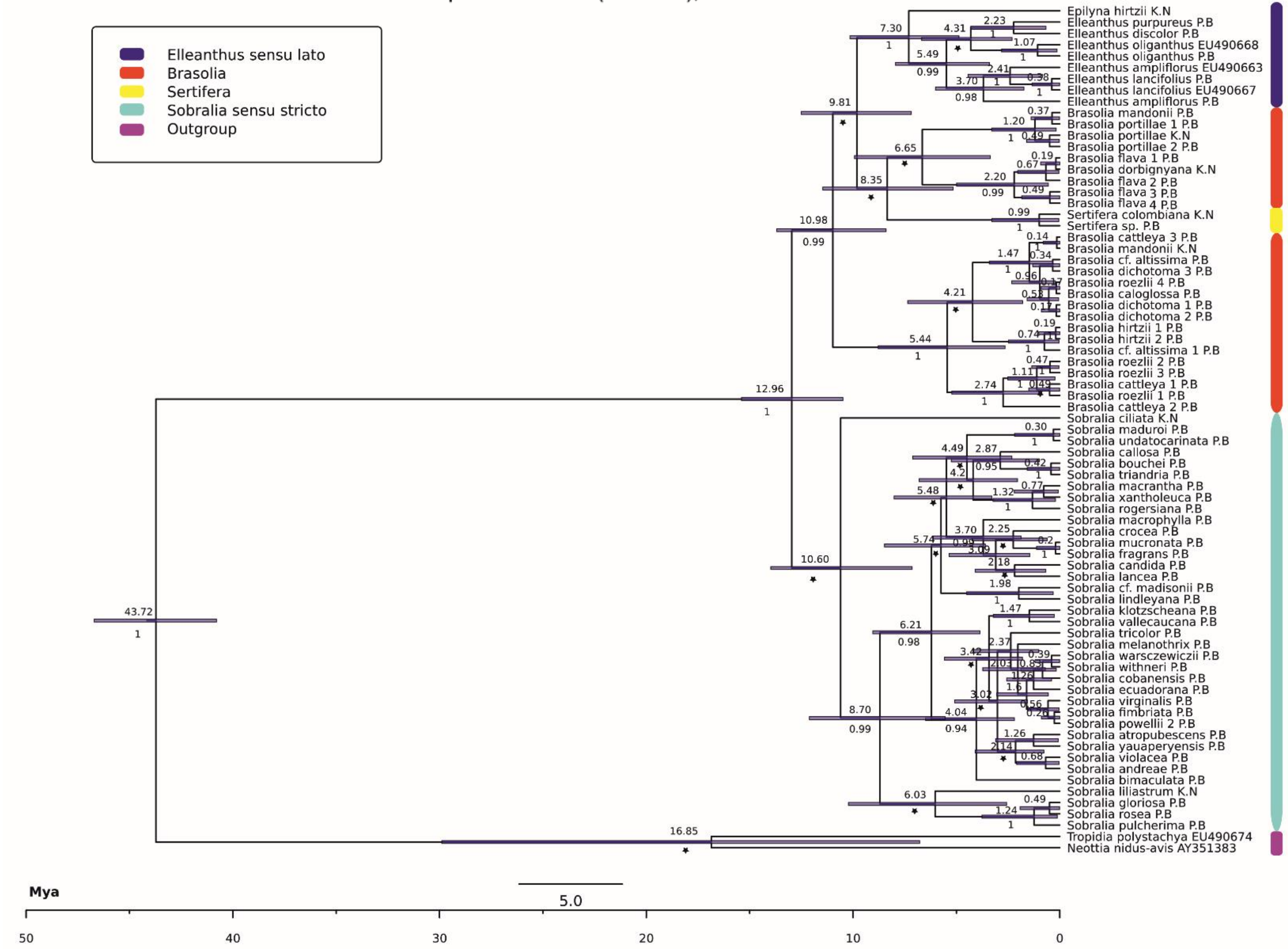

Supplementary material Table S1. List of samples used in the analysis with data on values of climatic variables and ecoregion.

Spec,Latitude,Longitude
Brasolia_cattleya,2.389011,-75.8942468999999
Brasolia_cattleya,3.2786111,-74.7977778
Brasolia_cattleya,4.32998,-73.8631829999999
Brasolia_cattleya,4.8166667,-76.1333333333333
Brasolia_cattleya,7.348395,-72.945739
Brasolia_cattleya,8.7461080744393,-68.37890625
Brasolia_ciliata,10.1,-64.0999999999999
Brasolia_ciliata,10.6833332999999,-62.6
Brasolia_ciliata,-4.0078909,-79.2112769
Brasolia_ciliata,-4.1,-78.5833333333333
Brasolia_ciliata,-4.2,-78.6833333333333
Brasolia_ciliata,-5.75,-77.7166666666666
Brasolia_ciliata,6.0763889,-73.1347222222222
Brasolia_dichotoma,-10.5469443999999,-75.3730555555555
Brasolia_dichotoma,-10.6766667,-75.3152777777777
Brasolia_dichotoma,-12.9086111,-72.8125
Brasolia_dichotoma,-13.1,-72.2166666666666
Brasolia_dichotoma,-13.1201994,-72.9763209999999
Brasolia_dichotoma,-13.1631412,-72.5449628999999
Brasolia_dichotoma,-13.1631412,-72.5449628999999
Brasolia_dichotoma,-13.1631412,-72.5449628999999
Brasolia_dichotoma,-13.1631412,-72.5449628999999
Brasolia_dichotoma,-13.3077235,-72.1143179
Brasolia_dichotoma,-14.3175114,-69.4678265
Brasolia_dichotoma,-14.3175114,-69.4678265
Brasolia_dichotoma,-16.3,-67.8166666666666
Brasolia_dichotoma,-16.6999999999999,-67.2333333333333

Brasolia_dichotoma,-17.4139766,-66.1653223999999
Brasolia_dichotoma,4.974252,-74.2913859999999
Brasolia_dichotoma,4.984372,-74.4603749999999
Brasolia_dichotoma,-5.75,-77.8666666666666
Brasolia_dichotoma,-5.8333333,-77.9630555555555
Brasolia_dichotoma,7.20182999999999,-72.966757
Brasolia_dichotoma,-9.7234965,-76.0973596
Brasolia_dichotoma,-9.7234965,-76.0973596
Brasolia_dorbignyana,-16.2166667,-67.7833333333333
Brasolia_dorbignyana,-16.25,-67.7833333333333
Brasolia_dorbignyana,-16.25,-67.7833333333333
Brasolia_dorbignyana,-16.25,-67.7833333333333
Brasolia_dorbignyana,-5,-78.8499999999999
Brasolia_dorbignyana,5.2427778,-76.0955555555555
Brasolia_rupicola,-17.7333333,-63.6499999999999
Brasolia_rupicola,-17.7666666999999,-63.6499999999999
Brasolia_rupicola,-18.1,-63.7999999999999
Brasolia_rupicola,-18.1772379,-63.8817831999999
Brasolia_speciosa,0.886389,-65.9894439999999
Brasolia_speciosa,1.4333333,-65.7833333333333
Brasolia_speciosa,1.5333333,-65.8166666666666
Brasolia_stenophylla,4.6064435,-61.1053690999999
Brasolia_stenophylla,4.8,-60.2666666666666
Brasolia_stenophylla,5.13157289999999,-60.7584603999999
Brasolia_stenophylla,5.13157289999999,-60.7584603999999
Brasolia_stenophylla,5.13157289999999,-60.7584603999999
Brasolia_stenophylla,5.13157289999999,-60.7584603999999
Brasolia_stenophylla,5.13157289999999,-60.7584603999999
Brasolia_stenophylla,5.13157289999999,-60.7584603999999
Brasolia_stenophylla,5.3471799,-61.7005649999999

Brasolia_stenophylla,5.7333333,-62.5333333333333
Brasolia_stenophylla,5.95,-61.383333
Elleanthus_ampliflorus,0.8733333,-78.4744444444444
Elleanthus_ampliflorus,4.7369444,-76.3002777777778
Elleanthus_ampliflorus,4.7575,-76.2363888888889
Elleanthus_ampliflorus,5.2497222,-76.1097222222222
Elleanthus_ampliflorus,-76.3166666666667,6.5333333
Elleanthus_lancifolius,1.1666667,-77.9666666667
Elleanthus_lancifolius,1.2833333,-78.1333333333
Elleanthus_lancifolius,1.8597222,-75.9752777778
Elleanthus_lancifolius,2.3463889,-76.2658333333
Elleanthus_lancifolius,3.4461111,-76.5466666667
Elleanthus_lancifolius,3.4858333,-76.6227777778
Elleanthus_lancifolius,3.5188889,-76.6991666667
Elleanthus_lancifolius,-4.1833333,-79.9463888889
Elleanthus_lancifolius,4.75,-76.2666666667
Elleanthus_lancifolius,4.7572222,-76.2075
Elleanthus_lancifolius,4.9716667,-76.2352777778
Elleanthus_lancifolius,4.9716667,-76.2352777778
Elleanthus_lancifolius,5.2441667,-76.0983333333
Elleanthus_lancifolius,5.2481944,-76.1038055556
Elleanthus_lancifolius,6.0636111,-75.2658333333
Sobralia_albolutea,10.3270781,-84.4356705999999
Sobralia_albolutea,10.3333333,-84.7166666666666
Sobralia_albolutea,10.4166667,-84.8833333333333
Sobralia_albolutea,10.4333332999999,-84.9166666666666
Sobralia_albolutea,10.8805555999999,-85.4013888888888
Sobralia_antioquiensis,2.452695,-76.811322
Sobralia_antioquiensis,3.4516467,-76.5319853999999
Sobralia_antioquiensis,4.44467599999999,-75.2424379999999

Sobralia_antioquiensis,4.44467599999999,-75.2424379999999
Sobralia_antioquiensis,5.19950499999999,-74.886835
Sobralia_bradeorum,10.3333333,-84.75
Sobralia_bradeorum,10.3666667,-85.8499999999999
Sobralia_bradeorum,10.4333332999999,-84.9
Sobralia_bradeorum,10.4563618999999,-84.9713512
Sobralia_bradeorum,10.4563618999999,-84.9713512
Sobralia_bradeorum,10.508849,-84.964356
Sobralia_bradeorum,10.8805555999999,-85.4013888888888
Sobralia_bradeorum,10.9125,-85.4722222222222
Sobralia_bradeorum,11.5333333,-85.6166666666666
Sobralia_bradeorum,11.8344444,-85.9783333
Sobralia_bradeorum,12.3,-85.4
Sobralia_bradeorum,15.6374999999999,-86.8611111111111
Sobralia_bradeorum,9.7722222,-83.8611111111111
Sobralia_bradeorum,9.80965059999999,-83.7041964
Sobralia_candida,-0.7166667,-77.6666666666666
Sobralia_candida,0.874452,-78.4678119999999
Sobralia_candida,-4.0333333,-78.9833333333333
Sobralia_candida,-4.0333333,-79
Sobralia_candida,-4.0833333,-78.9166666666666
Sobralia_candida,-6.65,-76.4166666666666
Sobralia_chrysostoma,10.0222222,-83.6638888888888
Sobralia_chrysostoma,10.2236110999999,-84.575
Sobralia_chrysostoma,10.3166667,-84.7333333333333
Sobralia_chrysostoma,10.5,-83.7833333333333
Sobralia_chrysostoma,10.6672221999999,-85.0947222222222
Sobralia_chrysostoma,8.75,-82.25
Sobralia_chrysostoma,9.9945605,-83.6763958
Sobralia_chrysostoma,9.9945605,-83.6763958

Sobralia_corazoi,0.810971,-77.920533
Sobralia_corazoi,0.9166667,-78.0999999999999
Sobralia_corazoi,10.05,-84.0583333333333
Sobralia_corazoi,4.8833333,-76.2166666666666
Sobralia_corazoi,8.7666667,-82.2999999999999
Sobralia_crocea,-0.1806532,-78.4678382
Sobralia_crocea,-0.1930117,-78.5000486999999
Sobralia_crocea,-0.2,-78.7833333333333
Sobralia_crocea,-0.2166667,-78.7999999999999
Sobralia_crocea,-0.2166667,-78.7999999999999
Sobralia_crocea,-0.421028,-78.791753
Sobralia_crocea,-0.4833333,-77.8666666666666
Sobralia_crocea,-0.65,-77.5
Sobralia_crocea,0.6691667,-77.5963888888888
Sobralia_crocea,-0.6691667,-77.5963888888888
Sobralia_crocea,-0.7166667,-77.8166666666666
Sobralia_crocea,-0.7166667,-77.8666666666666
Sobralia_crocea,0.810971,-77.920533
Sobralia_crocea,0.874452,-78.4678119999999
Sobralia_crocea,0.9,-78.0999999999999
Sobralia_crocea,0.9,-78.0999999999999
Sobralia_crocea,-1,-75.4666666666666
Sobralia_crocea,-1.4026177,-78.1898957999999
Sobralia_crocea,-1.4333333,-78.0999999999999
Sobralia_crocea,-1.4574523,-78.1079763
Sobralia_crocea,-1.4574523,-78.1079763
Sobralia_crocea,-10.2166666999999,-75.2666666666666
Sobralia_crocea,-10.2886111,-75.5183333333333
Sobralia_crocea,-10.6666667,-75.3333333333333
Sobralia_crocea,-10.6680556,-75.3194444444444

Sobralia_crocea,-10.75,-75.3666666666666
Sobralia_crocea,-2.3519444,-78.0349999999999
Sobralia_crocea,-3.4666667,-78.25
Sobralia_crocea,3.52431,-76.712851
Sobralia_crocea,-3.956916,-79.0469949999999
Sobralia_crocea,-3.9833333,-79.0666666666666
Sobralia_crocea,-3.9833333,-79.1333333333333
Sobralia_crocea,-4.5408333,-79.1305555555555
Sobralia_crocea,5.39978,-75.91613
Sobralia_crocea,-5.6833333,-77.65
Sobralia_crocea,-5.7025,-77.885
Sobralia_crocea,-5.75,-77.4833333333333
Sobralia_crocea,7.0772222,-75.4680556
Sobralia_decora,10.4833333,-84.6833333333333
Sobralia_decora,10.9833333,-85.1166666666666
Sobralia_decora,11.4458333,-85.5111111111111
Sobralia_decora,11.5416667,-85.5861111111111
Sobralia_decora,11.8166667,-85.9666666666666
Sobralia_decora,11.9,-84.9833333333333
Sobralia_decora,11.9852778,-86.1608332999999
Sobralia_decora,11.9852778,-86.1608332999999
Sobralia_decora,13.65,-84.7999999999999
Sobralia_decora,13.6999999999999,-84.8333333333333
Sobralia_decora,13.6999999999999,-85.8333333333333
Sobralia_decora,14,-84.1666666666666
Sobralia_decora,14.5704198,-89.336925
Sobralia_decora,14.9817467,-87.8893382999999
Sobralia_decora,15.4650586,-90.3842526999999
Sobralia_decora,15.6668524,-90.4276121999999
Sobralia_decora,15.6668524,-90.4276121999999

Sobralia_decora,15.6668524,-90.4276121999999
Sobralia_decora,15.7732601,-87.4653501999999
Sobralia_decora,15.7732601,-87.4653501999999
Sobralia_decora,15.7732601,-87.4653501999999
Sobralia_decora,15.7732601,-87.4653501999999
Sobralia_decora,16.6758333,-91.0347222222222
Sobralia_decora,16.7622,-91.0072
Sobralia_decora,16.7622,-91.0072
Sobralia_decora,16.7622,-91.0072
Sobralia_decora,16.7622,-91.0072
Sobralia_decora,16.7622,-91.0072
Sobralia_decora,16.7622,-91.0072
Sobralia_decora,16.7622,-91.0072
Sobralia_decora,16.7622,-91.0072
Sobralia_decora,16.78,-91.1205555555555
Sobralia_decora,16.8999999999999,-94.5083333333333
Sobralia_decora,16.9333333,-94.675
Sobralia_decora,16.9513889,-91.2619444444444
Sobralia_decora,17.0045593,-88.791402
Sobralia_decora,17.2666666999999,-94.6166666666666
Sobralia_decora,17.2937632999999,-93.0083015
Sobralia_decora,17.949192,-94.9145973999999
Sobralia_decora,-2.75,-60.9166666666666
Sobralia_decora,2.9683333,-78.1844443999999
Sobralia_decora,3.516667,-76.716667
Sobralia_decora,32.2547222,-116.531944399999
Sobralia_decora,7.96652379999999,-81.3289396999999
Sobralia_decora,7.9666667,-78.3833333333333
Sobralia_decora,8.25,-79.1333333333333
Sobralia_decora,8.6097836,-80.1316927

Sobralia_decora,8.69999999999999,-83.5166666666666
Sobralia_decora,8.7871912,-79.5575341999999
Sobralia_decora,8.96139619999999,-79.5631536999999
Sobralia_decora,8.96139619999999,-79.5631536999999
Sobralia_decora,9.1164638,-79.6965129999999
Sobralia_decora,9.11754631367052,-79.6976566314697
Sobralia_decora,9.2983333,-83.7722222222222
Sobralia_decora,9.2983333,-83.7722222222222
Sobralia_ecuadorana,0.35,-79.7333333333333
Sobralia_ecuadorana,-0.4144858,-78.7993261999999
Sobralia_ecuadorana,-0.4144858,-78.7993261999999
Sobralia_ecuadorana,-0.4144858,-78.7993261999999
Sobralia_ecuadorana,0.810971,-77.920533
Sobralia_ecuadorana,0.810971,-77.920533
Sobralia_elisabethae,0.6213889,-69.7489999999999
Sobralia_elisabethae,-0.633333,-72.25
Sobralia_elisabethae,-0.75,-72.3333333333333
Sobralia_elisabethae,0.82925,-70.0640555555555
Sobralia_elisabethae,-0.9666667,-69.75
Sobralia_elisabethae,-1.0833333,-71.1666666666666
Sobralia_elisabethae,1.1944444,-70.2399999999999
Sobralia_elisabethae,1.2,-70.2333333333333
Sobralia_elisabethae,1.2521739,-70.2336179999999
Sobralia_elisabethae,-1.2969444,-69.6174999999999
Sobralia_elisabethae,-1.2969444,-69.6174999999999
Sobralia_elisabethae,2.29975,-72.0546899
Sobralia_elisabethae,2.4722222,-72.6916666666666
Sobralia_elisabethae,2.4744444,-72.6966666666666
Sobralia_elisabethae,2.8852778,-65.7275
Sobralia_elisabethae,3.6333333,-66.8666666666666

Sobralia_elisabethae,3.6556389,-67.3945277777777
Sobralia_elisabethae,3.6666667,-65.75
Sobralia_elisabethae,3.8166667,-65.7
Sobralia_elisabethae,5.0333333,-60.6499999999999
Sobralia_elisabethae,5.1333333,-60.9833333333333
Sobralia_elisabethae,5.65,-61.1499999999999
Sobralia_elisabethae,5.7666667,-61.35
Sobralia_fenzliana,-0.5,-78
Sobralia_fenzliana,-0.65,-79.0833333333333
Sobralia_fenzliana,-1.0333333,-80.6833333333333
Sobralia_fenzliana,1.2521739,-70.2336179999999
Sobralia_fenzliana,-1.2883333,-77.9333333333333
Sobralia_fenzliana,11.05,-84.45
Sobralia_fenzliana,12.8333333,-85
Sobralia_fenzliana,13.6333333,-84.7999999999999
Sobralia_fenzliana,13.65,-84.8166666666666
Sobralia_fenzliana,14.6833332999999,-84.0666666666666
Sobralia_fenzliana,-3.9369444,-78.6241666666666
Sobralia_fragrans,0,-76.2
Sobralia_fragrans,-0.7333333,-76.8666666666666
Sobralia_fragrans,-0.9166667,-76.1833333333333
Sobralia_fragrans,1.0833333,-72.6666666666666
Sobralia_fragrans,1.0833333,-72.6666666666666
Sobralia_fragrans,1.3833333,-58.9333333333333
Sobralia_fragrans,-1.4574523,-78.1079763
Sobralia_fragrans,10.2133499,-83.7868871
Sobralia_fragrans,10.35,-84.6666666666666
Sobralia_fragrans,10.75,-83.5999999999999
Sobralia_fragrans,10.9166667,-84.2833333333333
Sobralia_fragrans,11.0166667,-84.2333333333333

Sobralia_fragrans,11.0333333,-84.4
Sobralia_fragrans,11.0333333,-84.4166666666666
Sobralia_fragrans,11.0411111,-84.3144444444444
Sobralia_fragrans,11.05,-84.4
Sobralia_fragrans,11.1,-83.9833333333333
Sobralia_fragrans,11.1208332999999,-84.3511111111111
Sobralia_fragrans,11.1208332999999,-84.3511111111111
Sobralia_fragrans,11.1208332999999,-84.3511111111111
Sobralia_fragrans,11.1208332999999,-84.3511111111111
Sobralia_fragrans,11.2333333,-85.15
Sobralia_fragrans,11.5666667,-84.0166666666666
Sobralia_fragrans,11.6,-83.6666666666666
Sobralia_fragrans,11.7833333,-84.7
Sobralia_fragrans,11.9499999999999,-84.2666666666666
Sobralia_fragrans,12.6630461,-84.7354741
Sobralia_fragrans,12.8333333,-85
Sobralia_fragrans,12.9833333,-85.9
Sobralia_fragrans,13.6452778,-85.1183333333333
Sobralia_fragrans,13.65,-84.4
Sobralia_fragrans,13.7666667,-84.7833333333333
Sobralia_fragrans,13.7666667,-84.9833333333333
Sobralia_fragrans,13.85,-85.3666666666666
Sobralia_fragrans,13.9666666999999,-84.2
Sobralia_fragrans,13.9666666999999,-84.2
Sobralia_fragrans,14.05,-84.6166666666666
Sobralia_fragrans,14.3666667,-84.5999997222222
Sobralia_fragrans,14.65,-84.0666666666666
Sobralia_fragrans,14.9817467,-87.8893382999999
Sobralia_fragrans,15.25,-85.4166666666666
Sobralia_fragrans,15.6668524,-90.4276121999999

Sobralia_fragrans,15.6668524,-90.4276121999999
Sobralia_fragrans,15.7732601,-87.4653501999999
Sobralia_fragrans,15.7732601,-87.4653501999999
Sobralia_fragrans,16.2000031999999,-89.4399387999999
Sobralia_fragrans,16.3333333,-91.1833333333333
Sobralia_fragrans,16.35,-91.2166666666666
Sobralia_fragrans,16.3999999999999,-91.2166666666666
Sobralia_fragrans,16.703927,-91.0646408999999
Sobralia_fragrans,16.7408332999999,-91.1102777777777
Sobralia_fragrans,16.7622,-91.0072
Sobralia_fragrans,16.7622,-91.0072
Sobralia_fragrans,16.7622,-91.0072
Sobralia_fragrans,16.7622,-91.0072
Sobralia_fragrans,16.7622,-91.0072
Sobralia_fragrans,16.7622,-91.0072
Sobralia_fragrans,16.7672221999999,-91.1083333333333
Sobralia_fragrans,16.7672221999999,-91.1083333333333
Sobralia_fragrans,16.8402777999999,-91.1422222222222
Sobralia_fragrans,16.9499999999999,-94.6833333333333
Sobralia_fragrans,17.0397302999999,-88.5202702999999
Sobralia_fragrans,2.6416369,-73.6005351
Sobralia_fragrans,2.69,-53.8580555555555
Sobralia_fragrans,2.7680556,-54.8541666666666
Sobralia_fragrans,3.1,-54.5499999999999
Sobralia_fragrans,3.1638889,-56.4611111111111
Sobralia_fragrans,3.6,-56.5
Sobralia_fragrans,3.6833333,-56.5
Sobralia_fragrans,3.6833333,-56.5
Sobralia_fragrans,3.6833333,-56.5
Sobralia_fragrans,3.75,-56.5

Sobralia_fragrans,3.75,-56.5
Sobralia_fragrans,4.083333,-56.2
Sobralia_fragrans,4.083333,-56.2
Sobralia_fragrans,4.7694444,-73.3238888888888
Sobralia_fragrans,5.1,-64.2
Sobralia_fragrans,5.6666667,-77.2666666666666
Sobralia_fragrans,5.7083333,-66.1333333333333
Sobralia_fragrans,6.15,-66.3833333333333
Sobralia_fragrans,6.5666667,-66.3833333333333
Sobralia_fragrans,7.4693076,-81.7568253
Sobralia_fragrans,7.5,-81.8166666666666
Sobralia_fragrans,7.75,-77.6666666666666
Sobralia_fragrans,7.7833333,-71.7666666666666
Sobralia_fragrans,8.5666667,-83.5166666666666
Sobralia_fragrans,8.6666667,-83.2
Sobralia_fragrans,8.75,-80.5833333333333
Sobralia_fragrans,8.96139619999999,-79.5631536999999
Sobralia_fragrans,9.3166667,-78.9166666666666
Sobralia_fragrans,9.80965059999999,-83.7041964
Sobralia_gloriosa,-0.2313889,-78.8027777777777
Sobralia_gloriosa,0.8666667,-78.1333333333333
Sobralia_gloriosa,0.8666667,-78.1333333333333
Sobralia_gloriosa,0.8666667,-78.1333333333333
Sobralia_gloriosa,0.874452,-78.4678119999999
Sobralia_gloriosa,3.5,-76.7
Sobralia_gloriosa,4.75,-76.3333333333333
Sobralia_gloriosa,4.8116667,-76.1711111111111
Sobralia_gloriosa,4.8166667,-76.15
Sobralia_granitica,2.1366111,-71.1614444444444
Sobralia_granitica,2.48021,-72.70055

Sobralia_granitica,3.45,-67.9666666666666
Sobralia_granitica,5.5183333,-67.5152777777777
Sobralia_granitica,5.75,-67.5
Sobralia_helleri,10.25,-84.1666666666666
Sobralia_helleri,10.3333333,-84.7166666666666
Sobralia_helleri,10.5833333,-83.5333333333333
Sobralia_helleri,10.7125,-84.9958333333333
Sobralia_helleri,10.9905556,-85.4277777777777
Sobralia_helleri,11.446,-85.5149999999999
Sobralia_infundibuligera,0.6544444,-70.2925
Sobralia_infundibuligera,0.7080556,-70.2372222222222
Sobralia_infundibuligera,0.8333333,-66.0833333333333
Sobralia_infundibuligera,0.85,-65.95
Sobralia_infundibuligera,-0.8666667,-69.75
Sobralia_infundibuligera,-0.8833333,-69.75
Sobralia_infundibuligera,-0.9666667,-69.75
Sobralia_infundibuligera,-0.9666667,-69.75
Sobralia_infundibuligera,-1.0352778,-71.5197222222222
Sobralia_infundibuligera,-1.3833333,-69.75
Sobralia_infundibuligera,3.6666667,-63
Sobralia_infundibuligera,4.8138889,-64.5694444444444
Sobralia_infundibuligera,5.13157289999999,-60.7584603999999
Sobralia_infundibuligera,5.1666667,-62.1166666666666
Sobralia_infundibuligera,5.3833333,-62.45
Sobralia_infundibuligera,5.8,-60.2333333333333
Sobralia_infundibuligera,5.8,-60.2333333333333
Sobralia_infundibuligera,5.8166667,-61.2333333333333
Sobralia_infundibuligera,5.85,-62.0333333333333
Sobralia_infundibuligera,5.9166667,-62.25
Sobralia_infundibuligera,5.9333333,-61.0833333333333

Sobralia_klotscheana,0.1138889,-78.6269444444444
Sobralia_klotscheana,-0.4166667,-78.75
Sobralia_klotscheana,-0.735415,-77.7864239999999
Sobralia_klotscheana,-0.75,-77.4666666666666
Sobralia_klotscheana,-1.4574523,-78.1079763
Sobralia_klotscheana,-1.6,-80.7
Sobralia_klotscheana,3.52431,-76.712851
Sobralia_klotscheana,3.57973999999999,-76.78173
Sobralia_lancea,0.0251731,-78.8920444
Sobralia_lancea,-0.1806532,-78.4678382
Sobralia_lancea,0.896133,-78.1148299999999
Sobralia_lancea,6.75,-76.3833333333333
Sobralia_leucoxantha,10.0333333,-83.9833333333333
Sobralia_leucoxantha,10.0333333,-84
Sobralia_leucoxantha,10.0583332999999,-84.0166666666666
Sobralia_leucoxantha,10.0708333,-83.9888888888888
Sobralia_leucoxantha,10.0708333,-83.9888888888888
Sobralia_leucoxantha,10.0910284,-84.4703933
Sobralia_leucoxantha,10.25,-84.7833333333333
Sobralia_leucoxantha,10.2749682,-84.8255096999999
Sobralia_leucoxantha,10.2758333,-84.7958333333333
Sobralia_leucoxantha,10.3,-84.4666666666666
Sobralia_leucoxantha,12.9166667,-84.6069444444444
Sobralia_leucoxantha,13.8166667,-89.9333333333333
Sobralia_leucoxantha,-15.43638,-68.360313
Sobralia_leucoxantha,9.3933333,-83.5891666666666
Sobralia_leucoxantha,9.5638889,-84.0541666666666
Sobralia_leucoxantha,9.69999999999999,-83.7833333333333
Sobralia_leucoxantha,9.71666669999999,-83.7833333333333
Sobralia_leucoxantha,9.7833333,-83.9666666666666

Sobralia_leucoxantha,9.7888889,-83.1383333333333
Sobralia_leucoxantha,9.86380909999999,-83.9161934999999
Sobralia_leucoxantha,9.9945605,-83.6763958
Sobralia_liliastrum,-0.2454485,-66.044194
Sobralia_liliastrum,0.3333333,-67.3333333333333
Sobralia_liliastrum,-0.3333333,-70.5
Sobralia_liliastrum,0.4333333,-72.5
Sobralia_liliastrum,-0.633333,-72.25
Sobralia_liliastrum,0.75,-70.5
Sobralia_liliastrum,0.9166667,-72.75
Sobralia_liliastrum,-0.9666667,-69.75
Sobralia_liliastrum,-0.9666667,-69.75
Sobralia_liliastrum,-0.9666667,-69.75
Sobralia_liliastrum,-1,-52
Sobralia_liliastrum,1.0833333,-72.6666666666666
Sobralia_liliastrum,1.25,-70.8499999999999
Sobralia_liliastrum,1.3730556,-70.9083333333333
Sobralia_liliastrum,1.4,-65.6333333333333
Sobralia_liliastrum,-1.5833333,-55.5333333333333
Sobralia_liliastrum,-1.5833333,-55.5333333333333
Sobralia_liliastrum,1.9,-67
Sobralia_liliastrum,1.9166667,-67.0833333333333
Sobralia_liliastrum,1.9333333,-67.0499999999999
Sobralia_liliastrum,1.95,-67.0499999999999
Sobralia_liliastrum,-12.4499999999999,-41.4166666666666
Sobralia_liliastrum,-12.4613888999999,-41.4211111111111
Sobralia_liliastrum,-12.4622221999999,-41.4199999999999
Sobralia_liliastrum,-12.4666666999999,-41.3666666666666
Sobralia_liliastrum,-12.5594444,-41.4183333333333
Sobralia_liliastrum,-12.8530555999999,-39.4886111111111

Sobralia_liliastrum,-14.8333333,-39.0333333333333
Sobralia_liliastrum,-14.9,-39.0333333333333
Sobralia_liliastrum,-15,-39.0499999999999
Sobralia_liliastrum,-15.1166667,-39.0333333333333
Sobralia_liliastrum,-15.1999999999999,-39.0499999999999
Sobralia_liliastrum,-17.2166667,-39.25
Sobralia_liliastrum,-17.2166667,-39.25
Sobralia_liliastrum,-18.8680556,-64.95
Sobralia_liliastrum,2.0833333,-60.1666666666666
Sobralia_liliastrum,2.1666667,-65.5666666666666
Sobralia_liliastrum,2.1666667,-65.5666666666666
Sobralia_liliastrum,-2.4722222,-72.6916666666666
Sobralia_liliastrum,2.566667,-72.6333329999999
Sobralia_liliastrum,2.566667,-72.6333329999999
Sobralia_liliastrum,3.0666667,-67.0999999999999
Sobralia_liliastrum,3.1333333,-65.6833333333333
Sobralia_liliastrum,3.55,-64.4833333333333
Sobralia_liliastrum,3.6666667,-65.75
Sobralia_liliastrum,3.6804288,-66.8022424
Sobralia_liliastrum,3.8166667,-65.7
Sobralia_liliastrum,4.3333333,-62.75
Sobralia_liliastrum,4.4,-67.7666666666666
Sobralia_liliastrum,4.5,-61.5
Sobralia_liliastrum,4.5,-61.5
Sobralia_liliastrum,4.5,-61.5833333333333
Sobralia_liliastrum,4.5666667,-65.5166666666666
Sobralia_liliastrum,4.5666667,-67.2999999999999
Sobralia_liliastrum,4.9,-67.5666666666666
Sobralia_liliastrum,5.17651599999999,-59.4807529
Sobralia_liliastrum,5.2,-67.8333333333333

Sobralia_liliastrum,5.3166667,-61.0666666666666
Sobralia_liliastrum,-5.3811326,-49.1331046
Sobralia_liliastrum,-5.3811326,-49.1331046
Sobralia_liliastrum,5.4,-67.5833333333333
Sobralia_liliastrum,5.5,-61.5833333333333
Sobralia_liliastrum,5.6166667,-67.3666666666666
Sobralia_liliastrum,5.6166667,-67.3666666666666
Sobralia_liliastrum,5.6666667,-60.2166666666666
Sobralia_liliastrum,5.6833333,-66.125
Sobralia_liliastrum,5.6916667,-66.125
Sobralia_liliastrum,5.75,-60.5833333333333
Sobralia_liliastrum,5.8,-60.2333333333333
Sobralia_liliastrum,5.85,-61.0955622222222
Sobralia_liliastrum,5.95,-60.6333330555555
Sobralia_liliastrum,5.9833333,-61.0499999999999
Sobralia_liliastrum,6.0452778,-60.6536111111111
Sobralia_liliastrum,-6.0666667,-50.1333333333333
Sobralia_liliastrum,6.5166667,-62.8833333333333
Sobralia_lindleyana,10.1325128,-84.3975671
Sobralia_lindleyana,10.1325128,-84.3975671
Sobralia_lindleyana,16.9087257999999,-92.0943350999999
Sobralia_lindleyana,8.5833333,-81.8333333333333
Sobralia_lindleyana,8.73216609999999,-82.6591465
Sobralia_lindleyana,8.73216609999999,-82.6591465
Sobralia_lindleyana,8.7833333,-82.6166666666666
Sobralia_lindleyana,8.8,-82.5833333333333
Sobralia_lindleyana,8.8972222,-82.7555555555555
Sobralia_lindleyana,8.9247222,-82.7963888888888
Sobralia_lindleyana,8.9427778,-82.81
Sobralia_lindleyana,9.4652778,-83.5597222222222

Sobralia_luerorum,-0.636498,-77.8036579999999
Sobralia_luerorum,-2.6333333,-78.549999999999
Sobralia_luerorum,7.1666667,-75.5
Sobralia_luteola,10.2133499,-83.7868871
Sobralia_luteola,11.0333333,-84.4
Sobralia_luteola,11.0333333,-84.4
Sobralia_luteola,11.0333333,-84.4166666666666
Sobralia_luteola,11.0663889,-84.3488888888888
Sobralia_luteola,11.0663889,-84.3488888888888
Sobralia_luteola,11.0663889,-84.3488888888888
Sobralia_luteola,11.1208332999999,-84.3511111111111
Sobralia_luteola,11.1666667,-84.4833333333333
Sobralia_luteola,11.5666667,-84.4333333333333
Sobralia_luteola,11.8166667,-84.3833333333333
Sobralia_luteola,11.9333332999999,-84.2999999999999
Sobralia_luteola,12.15,-84.2833333333333
Sobralia_luteola,12.8333333,-85
Sobralia_luteola,13.2833333,-85.4
Sobralia_luteola,13.9666666999999,-84.2
Sobralia_luteola,8.65416669999999,-83.1680555555555
Sobralia_luteola,9.19999999999999,-83.4166666666666
Sobralia_luteola,9.25,-83.4666666666666
Sobralia_macrantha,10.0910284,-84.4703933
Sobralia_macrantha,13.0333333,-86.3166666666666
Sobralia_macrantha,13.5174749999999,-86.8032828999999
Sobralia_macrantha,14,-87
Sobralia_macrantha,14,-87
Sobralia_macrantha,14.219958,-87.06815
Sobralia_macrantha,14.219958,-87.0683669999999
Sobralia_macrantha,14.219958,-87.0683669999999

Sobralia_macrantha,14.3131799999999,-90.2779459999999
Sobralia_macrantha,14.3131799999999,-90.2779459999999
Sobralia_macrantha,14.5666667,-90.5
Sobralia_macrantha,14.8166667,-89.3833333333333
Sobralia_macrantha,15.3691977,-92.2458096
Sobralia_macrantha,15.5073942,-92.1088215999999
Sobralia_macrantha,16.1666667,-96.9833333333333
Sobralia_macrantha,16.9087257999999,-92.0943350999999
Sobralia_macrantha,16.9325,-91.2619444444444
Sobralia_macrantha,16.9833333,-88.8
Sobralia_macrantha,16.9833333,-88.8
Sobralia_macrantha,17.0045593,-88.791402
Sobralia_macrantha,17.1666667,-95.8333333333333
Sobralia_macrantha,17.2666666999999,-96.0333333333333
Sobralia_macrantha,17.7192799,-92.8111933999999
Sobralia_macrantha,17.8833333,-96.65
Sobralia_macrantha,17.9166667,-96.65
Sobralia_macrantha,17.9499999999999,-96.6666666666666
Sobralia_macrantha,17.9666667,-96.6666666666666
Sobralia_macrantha,18.0333332999999,-96.6166666666666
Sobralia_macrantha,18.0666666999999,-96.8166666666666
Sobralia_macrantha,18.1999999999999,-96.75
Sobralia_macrantha,18.3125,-94.7166666666666
Sobralia_macrantha,18.8504744,-97.1036396
Sobralia_macrantha,18.8504744,-97.1036396
Sobralia_macrantha,18.8504744,-97.1036396
Sobralia_macrantha,18.8504744,-97.1036396
Sobralia_macrantha,18.8504744,-97.1036396
Sobralia_macrantha,19.9405934999999,-97.2956376999999
Sobralia_macrantha,27.9629897,-111.3769118

Sobralia_macrophylla,-0.0416667,-70.945
Sobralia_macrophylla,0.0833333,-70.6666666666666
Sobralia_macrophylla,-0.5,-77.3333333333333
Sobralia_macrophylla,-0.65,-77.3666666666666
Sobralia_macrophylla,0.8,-78.9
Sobralia_macrophylla,-1.0666667,-77.5999999999999
Sobralia_macrophylla,-1.0725,-69.5174722222222
Sobralia_macrophylla,1.4166667,-58
Sobralia_macrophylla,-10.25,-75.25
Sobralia_macrophylla,2.2666667,-54.5166666666666
Sobralia_macrophylla,2.9683333,-78.1844443999999
Sobralia_macrophylla,2.9683333,-78.1844443999999
Sobralia_macrophylla,2.9683333,-78.1844443999999
Sobralia_macrophylla,3.925,-56.1972222222222
Sobralia_macrophylla,-4.39728379999999,-59.7401488
Sobralia_macrophylla,-5.0555556,-78.3397222222222
Sobralia_macrophylla,5.8833333,-74.65
Sobralia_macrophylla,6,-74.75
Sobralia_macrophylla,6.569178,-76.8948899999999
Sobralia_macrophylla,8.58489799999999,-82.3885783
Sobralia_macrophylla,8.96139619999999,-79.5631536999999
Sobralia_mucronata,10.2133499,-83.7868871
Sobralia_mucronata,10.508849,-84.964356
Sobralia_mucronata,10.508849,-84.964356
Sobralia_mucronata,14.9817467,-87.8893382999999
Sobralia_mucronata,15.6668524,-90.4276121999999
Sobralia_mucronata,8.60009999999999,-80.1251
Sobralia_mucronata,8.6097836,-80.1316927
Sobralia_powellii,0.35,-79.7333333333333
Sobralia_powellii,-0.5737034,-79.3704908999999

Sobralia_powellii,-0.8833333,-79.1666666666666
Sobralia_powellii,-3.3259044,-79.8056660999999
Sobralia_powellii,-3.6252778,-78.4405555555555
Sobralia_powellii,8.5933178,-80.4465594
Sobralia_powellii,8.8230556,-80.6597222222222
Sobralia_powellii,8.8275,-80.6744444444444
Sobralia_powellii,8.8694444,-80.6666666666666
Sobralia_powellii,8.9286111,-80.68
Sobralia_powellii,9.94999999999999,-83.4166666666666
Sobralia_pulcherrima,0,-78.6666666666666
Sobralia_pulcherrima,-0.1918103,-78.5018595
Sobralia_pulcherrima,0.874452,-78.4678119999999
Sobralia_pulcherrima,0.896133,-78.1148299999999
Sobralia_pulcherrima,0.896133,-78.1148299999999
Sobralia_pulcherrima,-3.63,-78.4472222222222
Sobralia_pulcherrima,3.63649079999999,-76.9115272
Sobralia_pulcherrima,3.6666667,-76.8333333333333
Sobralia_pulcherrima,4.1666667,-77.1666666666666
Sobralia_pulcherrima,5.7666667,-76.3333333333333
Sobralia_pulcherrima,5.8333333,-76.3333333333333
Sobralia_rosea,0.27526,-76.6377999999999
Sobralia_rosea,0.810971,-77.920533
Sobralia_rosea,-0.85,-79.0999999999999
Sobralia_rosea,0.874452,-78.4678119999999
Sobralia_rosea,0.874452,-78.4678119999999
Sobralia_rosea,-0.9166667,-78.9166666666666
Sobralia_rosea,0.9166667,-79.1833333333333
Sobralia_rosea,-0.9166667,-79.1833333333333
Sobralia_rosea,-1.0666667,-77.5999999999999
Sobralia_rosea,1.15234,-76.6510549999999

Sobralia_rosea,1.1666667,-76.5666666666666
Sobralia_rosea,-1.241443,-77.88266
Sobralia_rosea,-1.3208333,-77.6864999999999
Sobralia_rosea,-1.3333333,-78.1666666666666
Sobralia_rosea,-1.398996,-78.361243
Sobralia_rosea,-1.4,-78.2
Sobralia_rosea,-1.4,-78.2
Sobralia_rosea,-1.4459005,-78.1258713999999
Sobralia_rosea,-1.4459005,-78.1258713999999
Sobralia_rosea,-1.45,-78.0999999999999
Sobralia_rosea,-1.45,-78.8333333333333
Sobralia_rosea,-1.4574523,-78.1079763
Sobralia_rosea,-1.4574523,-78.1079763
Sobralia_rosea,-1.5,-77.9333333333333
Sobralia_rosea,10.75,-74.3833333333333
Sobralia_rosea,-13.5911247,-70.9769244999999
Sobralia_rosea,2.452695,-76.811322
Sobralia_rosea,3.57973999999999,-76.78173
Sobralia_rosea,3.8830471,-77.0197211999999
Sobralia_rosea,-4.0333333,-78.9833333333333
Sobralia_rosea,-4.9166667,-78.3166666666666
Sobralia_rosea,-5.3,-78.3333333333333
Sobralia_rosea,-6.0419882,-76.9700669
Sobralia_rosea,6.554824,-73.1341199999999
Sobralia_rosea,-8.01486799999999,-76.659081
Sobralia_sessilis,0.0833333,-70.6666666666666
Sobralia_sessilis,-11.6280556,-41.0011111111111
Sobralia_sessilis,-11.6333333,-41.0333333333333
Sobralia_sessilis,-11.6333333,-41.0333333333333
Sobralia_sessilis,15.5942883,-90.1494988

Sobralia_sessilis,-16.1941783,-67.7288125
Sobralia_sessilis,2.1666667,-58.6666666666666
Sobralia_sessilis,2.8166667,-53.3666666666666
Sobralia_sessilis,3.1666667,-58.6666666666666
Sobralia_sessilis,3.3333333,-56.8166666666666
Sobralia_sessilis,-3.7172888,-79.6109048999999
Sobralia_sessilis,4.05,-64.3666666666666
Sobralia_sessilis,4.7666667,-63.2999999999999
Sobralia_sessilis,5.6384329,-54.0521131
Sobralia_sessilis,6.0833333,-62
Sobralia_sessilis,7.85,-59.2833333333333
Sobralia_setigera,-0.7166667,-77.5999999999999
Sobralia_setigera,-0.7166667,-77.6666666666666
Sobralia_setigera,0.874452,-78.4678119999999
Sobralia_setigera,-1.3928344,-78.4268758
Sobralia_setigera,-10.2166666999999,-75.5499999999999
Sobralia_setigera,-6.20841929999999,-77.6686449999999
Sobralia_setigera,7.119349,-73.1227415999999
Sobralia_setigera,8.9166667,-71.6833333333333
Sobralia_suaveolens,0.874452,-78.4678119999999
Sobralia_suaveolens,0.874452,-78.4678119999999
Sobralia_suaveolens,0.9666667,-78.4666666666666
Sobralia_suaveolens,1.565,-59.2383333333333
Sobralia_suaveolens,-2.4166667,-59.8999999999999
Sobralia_suaveolens,3.5333333,-53.2
Sobralia_suaveolens,3.6166667,-53.2
Sobralia_suaveolens,3.6333333,-53.2
Sobralia_suaveolens,5,-53.0249999999999
Sobralia_suaveolens,5.1083333,-59.9833333333333
Sobralia_suaveolens,7.4693076,-81.7568253

Sobralia_suaveolens,8.96139619999999,-79.5631536999999
Sobralia_suaveolens,8.96139619999999,-79.5631536999999
Sobralia_valida,1.202514,-76.9212249999999
Sobralia_valida,1.3166667,-58.95
Sobralia_valida,-1.4574523,-78.1079763
Sobralia_valida,-1.4574523,-78.1079763
Sobralia_valida,-1.4574523,-78.1079763
Sobralia_valida,1.7113889,-75.7144444444444
Sobralia_valida,-14.5,-67.4833333333333
Sobralia_valida,2.2166667,-68.2333333333333
Sobralia_valida,3.3333333,-59.25
Sobralia_valida,3.52431,-76.712851
Sobralia_valida,-3.680412,-79.6825274999999
Sobralia_valida,-3.9,-78.4249999999999
Sobralia_valida,3.9088889,-56.2122222222222
Sobralia_valida,3.95,-58.85
Sobralia_valida,3.95,-58.85
Sobralia_valida,3.95,-58.85
Sobralia_valida,4.7666667,-63.2999999999999
Sobralia_valida,4.7666667,-63.3166666666666
Sobralia_valida,4.9,-60.0833333333333
Sobralia_valida,4.90333,-77.339399
Sobralia_valida,5.0666667,-59.95
Sobralia_valida,5.15,-59.1333333333333
Sobralia_valida,5.15,-59.4833333333333
Sobralia_valida,5.1658333,-59.7777777777777
Sobralia_valida,5.1666667,-59.4833333333333
Sobralia_valida,5.17651599999999,-59.4807529
Sobralia_valida,5.17651599999999,-59.4807529
Sobralia_valida,5.1833333,-59.4833333333333

Sobralia_valida,5.1833333,-59.4833333333333
Sobralia_valida,5.1833333,-59.4833333333333
Sobralia_valida,5.1838889,-59.4825
Sobralia_valida,5.1838889,-59.4825
Sobralia_valida,5.2166667,-59.1
Sobralia_valida,5.2166667,-59.1
Sobralia_valida,5.3013889,-59.8977777777777
Sobralia_valida,5.3016667,-59.835
Sobralia_valida,5.3344444,-59.9249999999999
Sobralia_valida,5.3786111,-59.4925
Sobralia_valida,5.45,-60.6333333333333
Sobralia_valida,5.5897222,-60.2169444444444
Sobralia_valida,5.6333333,-60.0666666666666
Sobralia_valida,5.6833333,-60.2166666666666
Sobralia_valida,5.7,-60.2166666666666
Sobralia_valida,5.7166667,-60.1333333333333
Sobralia_valida,5.7344444,-63.6855555555555
Sobralia_valida,5.7666667,-60.25
Sobralia_valida,5.8,-60.2333333333333
Sobralia_valida,5.8166667,-61.1666666666666
Sobralia_valida,5.85,-61.0499999999999
Sobralia_valida,6.2477778,-60.4313888888888
Sobralia_violacea,0.0711111,-72.4466666666666
Sobralia_violacea,-1.031838,-77.7309569999999
Sobralia_violacea,-1.4923925,-78.0024134
Sobralia_violacea,-10.42,-75.5541666666666
Sobralia_violacea,10.8292413,-73.6923443
Sobralia_violacea,10.9337443,-85.4482484
Sobralia_violacea,-16.1812094,-67.750575
Sobralia_violacea,-16.1941783,-67.7288125

Sobralia_violacea,-16.35,-67.616667
Sobralia_violacea,2.6666667,-76.5666666666666
Sobralia_violacea,2.676697,-76.5331279999999
Sobralia_violacea,2.75391199999999,-76.6281139999999
Sobralia_violacea,3.06673,-76.23336
Sobralia_violacea,3.448069,-76.5394301999999
Sobralia_violacea,3.658519,-76.6908458999999
Sobralia_violacea,4.743647,-73.5349889999999
Sobralia_violacea,-4.9166667,-78.3166666666666
Sobralia_violacea,4.963945,-74.4340419999999
Sobralia_violacea,5.695633,-76.6498119999999
Sobralia_violacea,6.471563,-75.164646
Sobralia_violacea,6.485702,-75.3938309999999
Sobralia_violacea,6.95,-68.4499999999999
Sobralia_violacea,7.00087,-75.2772899
Sobralia_violacea,7.119349,-73.1227415999999
Sobralia_violacea,7.3666667,-59.7
Sobralia_violacea,8.1338118,-71.9786626
Sobralia_violacea,8.34061099999999,-71.466789
Sobralia_violacea,8.5698244,-71.1804988
Sobralia_violacea,8.5698244,-71.1804988
Sobralia_violacea,8.57536739999999,-71.3438398999999
Sobralia_violacea,9.1275891,-70.7094127
Sobralia_violacea,9.25344379999999,-70.2497615999999
Sobralia_violacea,9.25344379999999,-70.2497615999999
Sobralia_warszewiczii,11.8344444,-85.9783333
Sobralia_warszewiczii,11.8344444,-85.9783333
Sobralia_warszewiczii,12.2651512,-85.0778885999999
Sobralia_warszewiczii,9.7888889,-84.5
Sobralia_wilsoniana,8.6097836,-80.1316927

Sobralia_wilsoniana,8.6097836,-80.1316927
Sobralia_wilsoniana,8.71253239999999,-79.9080756999999
Sobralia_wilsoniana,9.3333333,-79.1333333333333
Sobralia_xantholeuca,14.5072221999999,-88.7849999999999
Sobralia_xantholeuca,15.4650586,-90.3842526999999
Sobralia_xantholeuca,15.4650586,-90.3842526999999
Sobralia_xantholeuca,16.1087415999999,-91.6991552
Sobralia_xantholeuca,16.7749999999999,-94.1583333333333

Supplementary material Table S2. Evaluation of created distribution models and estimates of relative contributions of the environmental variables to particular models.

Genus	Species	Group	AUC	var1	var2	var3
Brasolia	cattleya	Brasolia	0.957 (SD=0.016)	bio1 (39,5%)	bio4 (24,8%)	bio2 (20,8%)
Brasolia	ciliata	Brasolia	0.992 (SD=0.004)	bio2 (39,6%)	bio4 (27,8%)	bio1 (15,6%)
Brasolia	dichotoma	Brasolia	0.998 (SD=0.001)	bio1 (24,1%)	bio4 (19,7%)	bio18 (18,6%)
Brasolia	dorbignyana	Brasolia	0.982 (SD=0.009)	bio18 (27%)	bio1 (26,8%)	bio2 (25,4%)
Brasolia	rupicola	Brasolia	0.984 (SD=0.006)	bio1 (46,3%)	bio2 (21,2%)	bio18 (12,9%)
Brasolia	speciosa	Brasolia	1.000 (SD=0.000)	bio4 (22,9%)	bio2 (22,3%)	bio14 (21,4%)
Brasolia	stenophylla	Brasolia	0.966 (SD=0.011)	bio1 (50,7%)	bio4 (16,8%)	bio12 (13,5%)
Sobralia	corazoi	Intermediae	0.982 (SD=0.007)	bio18 (45,3%)	bio2 (20,2%)	bio4 (19,1%)
Sobralia	crocea	Intermediae	0.997 (SD=0.001)	bio14 (46,2%)	bio1 (21,3%)	bio4 (20,0%)
Sobralia	lancea	Intermediae	0.989 (SD=0.006)	bio4 (37,2%)	bio2 (24,8%)	bio1 (18,9%)
Sobralia	mucronata	Intermediae	0.983 (SD=0.010)	bio1 (63,2%)	bio12 (31,1%)	bio19 (2,3%)

Sobralia	suaveolens	Intermediae	0.992 (SD=0.003)	bio1 (57,0%)	bio19 (18,4%)	bio2 (8,7%)
Sobralia	valida	Intermediae	0.995 (SD=0.001)	bio12 (33,1%)	bio14 (31,1%)	bio1 (12,6%)
Sobralia	fragrans		0.993 (SD=0.004)	bio12 (53,1%)	bio19 (23,0%)	bio4 (7,1%)
Sobralia	wilsoniana		1.000 (SD=0.000)	bio1 (36,5%)	bio14 (17,2%)	bio19 (17,0%)
Sobralia	elisabethae	Racemosae	0.994 (SD=0.001)	bio12 (51,5%)	bio19 (21,6%)	bio14 (13,6%)
Sobralia	gloriosa	Racemosae	0.994 (SD=0.002)	bio4 (30,4%)	bio18 (25,7%)	bio2 (17,4%)
Sobralia	granitica	Racemosae	0.998 (SD=0.001)	bio19 (46,8%)	bio1 (41,5%)	bio14 (5,3%)
Sobralia	liliastrum	Racemosae	0.990 (SD=0.001)	bio14 (31,2%)	bio19 (25,6%)	bio1 (19,9%)
Sobralia	luteola	Racemosae	0.999 (SD=0.000)	bio12 (54,7%)	bio4 (17,2%)	bio14 (10,6%)
Sobralia	pulcherrima	Racemosae	0.997 (SD=0.001)	bio2 (20,4%)	bio4 (19,0%)	bio19 (17,9%)
Sobralia	rosea	Racemosae	0.995 (SD=0.001)	bio14 (24,6%)	bio4 (17,0%)	bio12 (15,7%)
Sobralia	albolutea	Sobralia1	1.000 (SD=0.000)	bio12 (64,5%)	bio14 (16,6%)	bio1 (8,2%)
Sobralia	decora	Sobralia1	0.993 (SD=0.002)	bio19 (40,4%)	bio12 (22,3%)	bio15 (20,6%)
Sobralia	fenzliana	Sobralia1	0.986 (SD=0.006)	bio1 (47,0%)	bio2 (16,6%)	bio12 (12,6%)
Sobralia	klotscheana	Sobralia1	0.982 (SD=0.013)	bio18 (32,1%)	bio1 (27,4%)	bio4 (18,4%)
Sobralia	sessilis	Sobralia1	0.965 (SD=0.010)	bio1 (78,4%)	bio4 (10,2%)	bio19 (3,9%)

Sobralia	violacea	Sobralia1	0.993 (SD=0.002)	bio12 (27,4%)	bio18 (19,8%)	bio4 (17,8%)
Sobralia	warszewiczii	Sobralia1	0.992 (SD=0.006)	bio1 (59,2%)	bio12 (10,9%)	bio14 (10,4%)
Sobralia	bradeorum	Sobralia2	0.997 (SD=0.002)	bio1 (45,8%)	bio12 (23,2%)	bio14 (6,7%)
Sobralia	chrysostoma	Sobralia2	0.999 (SD=0.001)	bio12 (74,1%)	bio14 (9,0%)	bio1 (5,7%)
Sobralia	ecuadorana	Sobralia2	0.987 (SD=0.012)	bio18 (49,5%)	bio4 (21,7%)	bio2 (17,2%)
Sobralia	helleri	Sobralia2	0.997 (SD=0.002)	bio12 (63,4%)	bio1 (18,7%)	bio14 (9,2%)
Sobralia	leucoxantha	Sobralia2	0.999 (SD=0.000)	bio12 (59,5%)	bio1 (19,5%)	bio4 (14,7%)
Sobralia	powellii	Sobralia2	0.984 (SD=0.008)	bio1 (52,1%)	bio18 (13,6%)	bio2 (11,5%)
Sobralia	setigera	Sobralia2	0.989 (SD=0.004)	bio1 (31,0%)	bio2 (26,6%)	bio4 (22,9%)
Sobralia	antioquiensis	Sobralia3	0.994 (SD=0.003)	bio1 (25,8%)	bio19 (21,1%)	bio2 (20,3%)
Sobralia	infundibuligera	Sobralia3	0.997 (SD=0.001)	bio14 (32,6%)	bio12 (25,0%)	bio18 (21,2%)
Sobralia	lindleyana	Sobralia3	0.995 (SD=0.004)	bio12 (56,7%)	bio14 (20,1%)	bio15 (14,3%)
Sobralia	macrantha	Sobralia3	0.996 (SD=0.001)	bio15 (38,4%)	bio18 (19,5%)	bio12 (11,2%)
Sobralia	macrophylla	Sobralia3	0.994 (SD=0.001)	bio19 (44,8%)	bio12 (21,3%)	bio14 (16,1%)
Sobralia	xantholeuca	Sobralia3	0.955 (SD=0.028)	bio1 (35,6%)	bio15 (18,7%)	bio18 (18,0%)
Sobralia	candida		0.994 (SD=0.002)	bio1 (32,4%)	bio2 (20,9%)	bio4 (20,0%)

Sobralia	luerorum	0.985 (SD=0.012)	bio19 (33,9%)	bio2 (23,2%)	bio4 (15,6%)

Supplementary material Table S3.

Niche overlap values calculated for all studied species. Values in bold received significant statistical support ($p<0.05$).

X	Brasolia_cattleya	Brasolia_ciliata	Brasolia_dichotoma	Brasolia_dorbignyana
Brasolia_ciliata	**0,63334183**			
Brasolia_dichotoma	**0,432456462**	**0,489693626**		
Brasolia_dorbignyana	0,454223228	**0,580664373**	**0,604367741**	
Brasolia_rupicola	0,090672087	**0,037369319**	0,201346073	**0,16651347**
Brasolia_speciosa	**2,22E-16**	**-2,22E-16**	**-2,22E-16**	**-2,22E-16**
Brasolia_stenophylla	**0,28340298**	**0,125679843**	**0,04840082**	0,225564741
Elleanthus_ampliflorus	0,098935615	-2,22E-16	0,002720348	**0,0141526**
Elleanthus_lancifolius	0,427037407	0,239750756	0,070177913	0,161912545
Sobralia_albolutea	**0,057396627**	**-2,22E-16**	**0,010791013**	**-2,22E-16**
Sobralia_antioquiensis	**0,187799677**	**-2,22E-16**	**0,024887272**	**-2,22E-16**
Sobralia_bradeorum	**0,307079186**	**0,272824102**	**0,384156175**	**0,326748586**
Sobralia_candida	0,634276491	0,442400976	**0,502016048**	**0,557628879**
Sobralia_chrysostoma	0,04231589	-2,22E-16	**0,014394553**	**0,010923616**
Sobralia_corazoi	**0,503726931**	0,426880669	0,216810698	**0,342622349**
Sobralia_crocea	0,633550697	0,534137107	**0,428241209**	**0,488068411**
Sobralia_decora	**0,427981285**	**0,317484601**	0,296196723	**0,397592862**
Sobralia_ecuadorana	0,468157563	0,343442078	0,296251323	**0,389637681**
Sobralia_elisabethae	0,061495231	0,007242533	**0,0190861**	**0,021613649**
Sobralia_fenzliana	0,563456367	0,455953899	**0,386138872**	**0,512601217**
Sobralia_fragrans	**0,371716988**	0,161079805	0,076268093	**0,18627737**
Sobralia_gloriosa	0,534057533	0,49938075	0,449372297	**0,445263615**
Sobralia_granitica	0,057337805	-2,22E-16	0,020792421	**0,002798512**
Sobralia_helleri	**0,20720666**	**0,083113307**	**0,027735978**	**0,064599928**
Sobralia_infundibuligera	**0,328216257**	**0,247028072**	0,101032677	**0,1971685**
Sobralia_klotscheana	**0,509156899**	**0,378352013**	0,371061843	**0,390589276**
Sobralia_lancea	**0,393512511**	**0,569460331**	**0,395947507**	**0,38576134**
Sobralia_leucoxantha	0,225133942	0,160063152	**0,1500813**	**0,22329764**
Sobralia_liliastrum	**0,390298671**	0,251957514	**0,261922281**	**0,274414369**
Sobralia_lindleyana	0,152930477	0,027389015	**0,023793292**	**0,158353691**
Sobralia_luerorum	**0,047246819**	0,034441992	**0,006202832**	**0,022565062**
Sobralia_luteola	0,203616747	0,017155457	**0,041101163**	**0,050681563**
Sobralia_macrantha	**0,121487141**	**0,067037065**	**0,299200868**	**0,379149381**
Sobralia_macrophylla	**0,377000915**	**0,275545949**	**0,116828453**	**0,198777281**
Sobralia_mucronata	0,492010458	**0,421440368**	0,217582622	0,347571219
Sobralia_powellii	0,379529181	0,199255636	0,109309752	0,202166839
Sobralia_pulcherrima	**0,460994717**	**0,206799818**	**0,305654118**	**0,475225567**
Sobralia_rosea	**0,470819613**	**0,342769073**	**0,278233423**	**0,435182783**
Sobralia_sessilis	**0,608053868**	**0,462372329**	0,468779471	**0,615244295**
Sobralia_setigera	**0,678517923**	**0,529594604**	**0,565939857**	**0,70263688**
Sobralia_suaveolens	**0,283410386**	**0,034209387**	0,057599964	0,054337446
Sobralia_valida	**0,609708066**	**0,514985334**	**0,328063348**	**0,421216675**
Sobralia_violacea	0,579849826	**0,395494632**	**0,424105805**	0,61762324
Sobralia_warszewiczii	0,507078055	**0,300239727**	0,544058017	**0,474058731**
Sobralia_wilsoniana	**0,124282942**	**-2,22E-16**	**0,054296119**	**0,052062288**
Sobralia_xantholeuca	**0,124715008**	**0,062562197**	**0,262755656**	0,189309893

Brasolia_rupicola	Brasolia_speciosa	Brasolia_stenophylla	Elleanthus_ampliflorus
**-2,22E-16**			
-2,22E-16	**-2,22E-16**		
**-2,22E-16**	0,180830634	**0,009532849**	
**0,001941015**	0,137700165	0,14797935	**0,400701962**
**-2,22E-16**	2,22E-16	**0,018942935**	0,181092118
**-2,22E-16**	-2,22E-16	**2,22E-16**	0,212990611
**0,365935456**	-2,22E-16	**0,228205859**	0,141077648
**0,097912061**	**-2,22E-16**	0,225330106	**0,100942248**
**-2,22E-16**	0,267247917	0,02374286	0,253947088
**2,22E-16**	2,22E-16	0,26474368	0,156615839
**0,086402584**	0,098849748	**0,251552792**	0,339225487
**0,108726966**	0,061085079	0,241726103	0,21048403
**0,004678628**	-2,22E-16	0,273226981	0,076173139
**-2,22E-16**	0,199762772	0,104715759	0,410573735
**0,108080998**	0,086002464	0,184105241	0,291219236
0,066720607	0,067022413	0,270880278	0,270999673
**0,019039751**	-2,22E-16	0,165976753	0,279589565
**-2,22E-16**	-2,22E-16	0,060051315	0,126617751
**-2,22E-16**	0,219354963	**0,153698511**	0,457806913
**0,059313637**	0,175495058	0,22850512	0,34863876
**0,077826985**	0,05712438	**0,165648602**	0,224981314
**0,045701888**	0,108202901	**0,145743002**	**0,277603394**
**0,121934397**	0,196463252	0,190917352	0,364408239
0,084860487	0,123247811	**0,205022683**	0,367337096
**0,050609685**	-2,22E-16	0,495149928	0,024597227
**-2,22E-16**	0,092084263	**-2,22E-16**	0,466706302
**0,022800295**	-2,22E-16	0,161613716	0,264523252
0,219250695	-2,22E-16	0,191589037	-2,22E-16
**0,0519306**	0,094440309	**0,129613719**	0,411398999
**0,134744782**	-2,22E-16	**0,328041074**	0,059587279
**0,081972652**	0,051132473	**0,182294358**	0,342230342
**0,087119536**	0,105857012	**0,167931903**	0,257930023
0,060133438	0,062943616	**0,133361514**	0,309933684
**0,119694117**	**0,122221427**	0,265036416	**0,190944162**
0,109524127	2,22E-16	**0,291866633**	0,073346001
-2,22E-16	0,046292186	0,273190385	0,351893468
**0,090008933**	**0,055931576**	0,275212919	0,229693043
**0,108476429**	**0,033558577**	0,231729806	0,261921976
**0,337458804**	-2,22E-16	**0,532555416**	-2,22E-16
**-2,22E-16**	-2,22E-16	**0,315608942**	**0,071997206**
0,387448856	**-2,22E-16**	0,097695931	0,023767481

Elleanthus_lancifolius	Sobralia_albolutea	Sobralia_antioquiensis	Sobralia_bradeorum
**0,209672507**			
**0,164015094**	**0,202196984**		
0,10938082	0,279991095	**0,132753697**	
**0,336997837**	**0,113651076**	**0,164616697**	**0,299377882**
0,579729738	0,338245761	0,100777377	**0,078522216**
**0,438571438**	0,226163514	0,24997797	**0,203785335**
0,501937643	**0,206933207**	0,153355473	**0,329974512**
**0,500893911**	**0,243952906**	0,174035566	**0,292169027**
0,217562776	0,135540848	0,09247207	0,184523455
**0,694389177**	**0,264713326**	0,067492589	0,080192704
0,678877525	0,20939179	0,204718183	0,275794794
0,611003373	0,303076422	0,185805375	0,26436792
**0,53032011**	**0,213409206**	0,199904749	0,300992293
**0,194222564**	**0,413613902**	0,090580543	**0,187164225**
**0,756823572**	**0,322206023**	0,164392078	**0,138101069**
**0,694707613**	**0,145546481**	**0,114750375**	0,221707457
**0,600899655**	**0,182168376**	0,124325753	**0,267502519**
**0,360104906**	**0,229215069**	0,014426074	**0,226404014**
**0,669520355**	**0,237087658**	0,08549824	**0,270476914**
0,753377049	0,253339431	0,181749328	**0,316948679**
0,217805855	0,103702928	0,027109152	**0,331112806**
0,472081086	0,297764249	0,037928476	**0,090987879**
**0,409511771**	**0,366170121**	0,28138093	**0,248782985**
0,091887713	0,003477335	-2,22E-16	**0,268542785**
0,628655285	0,213199824	0,166173919	**0,208673674**
**0,396637588**	**0,079281735**	**0,184232704**	**0,315362622**
**0,611947715**	**0,321890494**	0,230265527	**0,235299277**
**0,526980468**	**0,128583221**	0,111007813	**0,223415535**
0,659148508	**0,214915344**	0,102814549	**0,223769353**
**0,528653073**	**0,21944539**	**0,149016071**	**0,313713582**
**0,381709494**	0,089105789	0,137793901	**0,316189379**
0,354240448	0,41852939	0,457925929	0,306351927
0,565524366	0,188410913	0,213723711	0,323259131
0,455893951	0,187255325	0,192202493	0,317602655
0,118789054	0,005880991	-2,22E-16	0,575704097
**0,09207699**	**0,103367815**	-2,22E-16	0,074898157
0,122698055	0,072045326	-2,22E-16	**0,332870464**

Sobralia_candida	Sobralia_chrysostoma	Sobralia_corazoi	Sobralia_crocea	Sobralia_decora
**0,141990409**				
0,589602635	**0,173617201**			
0,649385202	**0,351273142**	**0,467682856**		
**0,550364136**	0,279616171	**0,507152535**	**0,543660125**	
0,555411697	0,067998773	0,640213663	0,424797035	**0,571447402**
0,079606633	**0,840833768**	**0,175212663**	**0,341052421**	0,269885257
0,649558561	0,382321845	0,53253511	**0,607244235**	0,803943005
0,550036204	0,422117248	0,567939442	**0,57625863**	0,742697201
0,688852971	0,258177931	**0,482949983**	**0,706528876**	0,44023648
0,197835315	**0,161335614**	0,420158474	**0,248355827**	0,382937433
**0,305323444**	**0,763869353**	**0,321866577**	**0,506285169**	**0,469156864**
0,329063241	0,694071144	0,316283579	**0,418534615**	0,489008709
0,641768927	0,306645891	**0,422796725**	0,72440786	0,679346257
**0,398617186**	**0,468003861**	**0,269810579**	**0,567719492**	**0,448290574**
0,417956221	0,699663506	0,294629714	0,488595456	0,596553589
0,603610516	0,489950975	0,495839284	0,636313623	0,664391766
0,27651662	0,088701996	0,306788112	**0,254821742**	0,364744814
0,11208006	0,669077002	0,199965176	**0,285048766**	0,250068667
0,36434335	**0,380849914**	0,585119568	**0,347921111**	0,588073194
0,358937629	0,0085614	0,168530164	**0,206917925**	0,515795248
0,503314976	0,561068829	0,46649505	**0,581802479**	0,581244349
**0,562612227**	0,23763825	0,554514846	0,51257111	0,72024634
0,568492791	**0,461848467**	0,428211806	0,549975312	0,729440871
0,553983129	**0,399632085**	0,371695303	**0,64134523**	0,529927878
0,603376105	**0,444486827**	0,474807903	**0,672904552**	0,707644622
**0,709145021**	**0,408459501**	**0,465001362**	**0,703165479**	**0,777202603**
**0,727520165**	0,080506803	0,5123607	0,680668212	**0,609166794**
0,357894634	0,247054773	0,574833423	0,386580767	0,436660237
0,64648997	0,341947845	0,595277166	0,644760841	0,807245188
0,667582754	0,290322035	0,505966909	0,479393123	0,811278294
0,388178235	0,014528707	0,183689698	0,324365192	0,365589963
0,116028134	0,071697127	0,184206552	0,125033809	0,159030256
0,32231106	0,037246295	0,293705087	0,23937127	0,406364469

Sobralia_ecuadorana	Sobralia_elisabethae	Sobralia_fenzliana	Sobralia_fragrans	Sobralia_gloriosa
**0,093777509**				
0,492255986	**0,348621064**			
0,530931551	0,496973844	**0,66519769**		
0,313614016	0,192380492	0,601054551	**0,423129833**	
0,529211625	0,241363838	0,216668975	0,353132078	**0,18081503**
**0,186308337**	**0,637604404**	**0,590030385**	**0,601158501**	**0,482733352**
0,301142775	**0,725792833**	0,473538433	0,61103159	0,271998786
**0,43685145**	0,453109598	**0,760759537**	**0,579539767**	**0,541168489**
**0,244137076**	0,419900538	**0,529515137**	**0,352722427**	**0,425488601**
0,339231634	0,667649486	0,61336522	**0,709009173**	0,384612571
0,407801566	0,645760711	0,743040211	0,737950576	0,504543478
0,431867133	0,110164273	0,266154171	0,420492225	0,173724972
0,040936101	0,465678223	0,290867035	0,40644254	0,267554948
**0,516284085**	0,317617118	0,560226666	**0,68166859**	**0,258337122**
**0,339159295**	0,037261649	0,505931732	0,482757114	0,081283042
**0,315832971**	0,545423833	0,698675325	**0,593637113**	0,481259796
**0,467432269**	**0,189157524**	**0,616728117**	**0,657881991**	**0,349046158**
0,442064322	0,370517469	0,705436846	**0,805359204**	**0,537566048**
**0,424767938**	0,406116704	0,730817163	**0,567733037**	**0,495667368**
**0,399147594**	0,578508711	0,774607523	**0,667461513**	**0,55956184**
**0,504700694**	**0,210953473**	**0,731435031**	**0,564441207**	**0,61460402**
**0,398232408**	**0,061270892**	0,658709655	0,38204121	**0,538783226**
0,308534442	0,252582913	0,398056005	0,466474566	0,482346784
0,565647073	0,371888103	0,736897063	0,695962414	0,618156644
0,459189317	0,224322409	0,8287801	0,610313445	0,6534645
0,218802234	0,055162215	0,308562053	0,229804706	0,254799038
0,168405114	0,083640704	0,119556293	0,172503159	0,122360745
0,308978722	0,088135413	0,260093716	0,311906034	0,122159252

	Sobralia_granitica	Sobralia_helleri	Sobralia_infundibuligera	Sobralia_klotscheana	Sobralia_lancea
	**0,227614905**				
	**0,265584667**	**0,43087396**			
	**0,162665166**	0,515955954	**0,536621285**		
	**0,046559867**	**0,478154188**	**0,370783339**	**0,427347946**	
	**0,215342624**	0,783667745	**0,688687408**	**0,558358569**	**0,46376522**
	0,265305805	**0,650152353**	**0,630823386**	0,637334656	0,517482443
	**0,305240502**	0,193201177	0,219264037	**0,23322038**	0,086984669
	**0,083398608**	**0,675029055**	**0,341013213**	**0,368634937**	0,514808016
	**0,546561211**	**0,636520846**	**0,293467497**	**0,40607303**	0,230376377
	**0,103378013**	**0,082848169**	**0,227768411**	**0,411462676**	**0,054428629**
	**0,202891926**	**0,58995547**	**0,631055649**	0,725835247	**0,454380529**
	**0,246953116**	**0,27205998**	0,467902591	**0,573025297**	**0,344444072**
	**0,280038934**	0,672495401	0,517828947	**0,632077813**	**0,433380134**
	**0,175707568**	0,427040978	**0,494716635**	**0,653812003**	0,415440888
	**0,209002392**	**0,613235473**	**0,655268515**	0,646016158	**0,481231261**
	**0,246628332**	**0,47499273**	**0,489892217**	**0,772776787**	**0,574766684**
	0,171324308	**0,234818299**	0,326663735	0,596077225	0,343185787
	0,485047998	0,480051223	0,230939121	0,309895348	0,115043418
	0,325010614	0,51188351	0,505843694	0,744638752	0,433945971
	0,244665191	0,501688794	0,425157907	0,758254895	0,425432826
	0,062275498	0,105146571	0,177341946	0,232064225	0,209817595
	0,200713726	0,09561078	0,097167275	0,085840459	-2,22E-16
	0,432001851	0,092823088	0,261132732	0,244265277	0,053119514

Sobralia_leucoxantha	Sobralia_liliastrum	Sobralia_lindleyana	Sobralia_luerorum	Sobralia_luteola
**0,708554749**				
0,300104169	**0,261888455**			
**0,511125109**	0,344525256	**0,076717277**		
0,488459323	0,546031638	0,483682025	**0,314413851**	
0,290912107	0,278409627	0,36722342	-2,22E-16	**0,202940443**
0,614721902	0,789747968	0,201443161	0,322288282	0,606933258
**0,37168529**	0,476317862	**0,361742441**	**0,045621287**	0,325944772
0,655835698	0,675911096	0,320052299	0,410342882	0,610962691
0,481192298	0,559149389	0,205734671	0,355773782	0,339198713
0,561875936	0,722712496	0,199472555	**0,295923163**	0,475207937
**0,57996072**	**0,692129104**	**0,297076803**	**0,271520001**	**0,511705369**
0,308232537	**0,468421298**	**0,233023213**	**0,058562155**	0,324209926
0,323902762	0,448926775	0,369955579	0,295225261	0,669546399
0,557506196	0,745946316	0,321419878	0,342355203	0,521203786
0,448939727	0,659278165	0,276254064	0,385042875	0,473389371
0,282472658	0,271649611	0,349704025	-2,22E-16	0,139392028
0,088720178	0,110995307	0,287030622	-2,22E-16	0,196744601
0,232385884	0,251248392	0,382304433	-2,22E-16	0,285984786

Sobralia_macrantha	Sobralia_macrophylla	Sobralia_mucronata	Sobralia_powellii
**0,281998105**			
**0,511801332**	**0,533010573**		
0,31417513	0,77351982	**0,547174396**	
0,337893498	**0,544348457**	0,559911931	**0,591091847**
0,313382676	**0,76556318**	0,515040596	0,680385196
**0,353273837**	**0,630298978**	**0,658628678**	**0,647342874**
0,375689153	**0,4310805**	0,643288721	0,503735222
0,107436235	0,369406071	0,298218052	0,454960819
0,430372406	0,76692548	0,726227723	0,756104087
0,455240661	0,50010603	0,502274251	0,670229231
0,426792791	0,1405024	0,295291345	0,182964997
0,092703101	0,097196169	0,154030048	0,115654684
0,416562848	0,149515126	0,297983473	0,261368409

Sobralia_pulcherrima	Sobralia_rosea	Sobralia_sessilis	Sobralia_setigera	Sobralia_suaveolens
**0,621286272**				
**0,621025191**	**0,659392841**			
0,554288104	**0,619963896**	**0,678849971**		
0,25044709	0,327471715	0,38327908	**0,319196369**	
0,478426064	0,783100835	0,761696142	**0,671250752**	**0,42049307**
0,662386779	0,597354654	0,778287603	0,746198636	**0,4496043**
0,236792894	0,187057972	0,376628396	0,417849411	**0,148386755**
0,097193817	0,075189336	0,175157998	**0,099591811**	**0,298573291**
0,199240946	0,202598565	0,325088649	**0,288308979**	**0,216215666**

Sobralia_valida	Sobralia_violacea	Sobralia_warszewiczii	Sobralia_wilsoniana
**0,790489027**			
0,360769868	**0,329558605**		
**0,1530884**	**0,103862658**	**0,18799558**	
**0,290566662**	0,256191718	0,387618492	**0,169205877**

X	Brasolia_cattleya	Brasolia_ciliata	Brasolia_dichotoma	Brasolia_dorbignyana
Brasolia_ciliata	**0,63334183**			
Brasolia_dichotoma	**0,432456462**	**0,489693626**		
Brasolia_dorbignyana	0,454223228	**0,580664373**	**0,604367741**	
Brasolia_rupicola	0,090672087	**0,037369319**	0,201346073	**0,16651347**
Brasolia_speciosa	**2,22E-16**	**-2,22E-16**	**-2,22E-16**	**-2,22E-16**
Brasolia_stenophylla	**0,28340298**	**0,125679843**	**0,04840082**	0,225564741
Elleanthus_ampliflorus	0,098935615	-2,22E-16	0,002720348	**0,0141526**
Elleanthus_lancifolius	0,427037407	0,239750756	0,070177913	0,161912545
Sobralia_albolutea	**0,057396627**	**-2,22E-16**	**0,010791013**	**-2,22E-16**
Sobralia_antioquiensis	**0,187799677**	**-2,22E-16**	**0,024887272**	**-2,22E-16**
Sobralia_bradeorum	**0,307079186**	**0,272824102**	**0,384156175**	**0,326748586**
Sobralia_candida	0,634276491	0,442400976	**0,502016048**	**0,557628879**
Sobralia_chrysostoma	0,04231589	-2,22E-16	**0,014394553**	**0,010923616**
Sobralia_corazoi	**0,503726931**	0,426880669	0,216810698	**0,342622349**
Sobralia_crocea	0,633550697	0,534137107	**0,428241209**	**0,488068411**
Sobralia_decora	**0,427981285**	**0,317484601**	0,296196723	**0,397592862**
Sobralia_ecuadorana	0,468157563	0,343442078	0,296251323	**0,389637681**
Sobralia_elisabethae	0,061495231	0,007242533	**0,0190861**	**0,021613649**
Sobralia_fenzliana	0,563456367	0,455953899	**0,386138872**	**0,512601217**
Sobralia_fragrans	**0,371716988**	0,161079805	0,076268093	**0,18627737**
Sobralia_gloriosa	0,534057533	0,49938075	0,449372297	**0,445263615**
Sobralia_granitica	0,057337805	-2,22E-16	0,020792421	**0,002798512**
Sobralia_helleri	**0,20720666**	**0,083113307**	**0,027735978**	**0,064599928**
Sobralia_infundibuligera	**0,328216257**	**0,247028072**	0,101032677	**0,1971685**
Sobralia_klotscheana	**0,509156899**	**0,378352013**	0,371061843	**0,390589276**
Sobralia_lancea	**0,393512511**	**0,569460331**	**0,395947507**	**0,38576134**
Sobralia_leucoxantha	0,225133942	0,160063152	**0,1500813**	**0,22329764**
Sobralia_liliastrum	**0,390298671**	0,251957514	**0,261922281**	**0,274414369**
Sobralia_lindleyana	0,152930477	0,027389015	**0,023793292**	**0,158353691**
Sobralia_luerorum	**0,047246819**	0,034441992	**0,006202832**	**0,022565062**
Sobralia_luteola	0,203616747	0,017155457	**0,041101163**	**0,050681563**
Sobralia_macrantha	**0,121487141**	**0,067037065**	**0,299200868**	**0,379149381**
Sobralia_macrophylla	**0,377000915**	**0,275545949**	**0,116828453**	**0,198777281**
Sobralia_mucronata	0,492010458	**0,421440368**	0,217582622	0,347571219
Sobralia_powellii	0,379529181	0,199255636	0,109309752	0,202166839
Sobralia_pulcherrima	**0,460994717**	**0,206799818**	**0,305654118**	**0,475225567**
Sobralia_rosea	**0,470819613**	**0,342769073**	**0,278233423**	**0,435182783**
Sobralia_sessilis	**0,608053868**	**0,462372329**	0,468779471	**0,615244295**
Sobralia_setigera	**0,678517923**	**0,529594604**	**0,565939857**	**0,70263688**
Sobralia_suaveolens	**0,283410386**	**0,034209387**	0,057599964	0,054337446
Sobralia_valida	**0,609708066**	**0,514985334**	**0,328063348**	**0,421216675**
Sobralia_violacea	0,579849826	**0,395494632**	**0,424105805**	0,61762324
Sobralia_warszewiczii	0,507078055	**0,300239727**	0,544058017	**0,474058731**
Sobralia_wilsoniana	**0,124282942**	**-2,22E-16**	**0,054296119**	**0,052062288**
Sobralia_xantholeuca	**0,124715008**	**0,062562197**	**0,262755656**	0,189309893

Brasolia_rupicola	Brasolia_speciosa	Brasolia_stenophylla	Elleanthus_ampliflorus
**-2,22E-16**			
-2,22E-16	**-2,22E-16**		
**-2,22E-16**	0,180830634	**0,009532849**	
**0,001941015**	0,137700165	0,14797935	**0,400701962**
**-2,22E-16**	2,22E-16	**0,018942935**	0,181092118
**-2,22E-16**	-2,22E-16	**2,22E-16**	0,212990611
**0,365935456**	-2,22E-16	**0,228205859**	0,141077648
**0,097912061**	**-2,22E-16**	0,225330106	**0,100942248**
**-2,22E-16**	0,267247917	0,02374286	0,253947088
**2,22E-16**	2,22E-16	0,26474368	0,156615839
**0,086402584**	0,098849748	**0,251552792**	0,339225487
**0,108726966**	0,061085079	0,241726103	0,21048403
**0,004678628**	-2,22E-16	0,273226981	0,076173139
**-2,22E-16**	0,199762772	0,104715759	0,410573735
**0,108080998**	0,086002464	0,184105241	0,291219236
0,066720607	0,067022413	0,270880278	0,270999673
**0,019039751**	-2,22E-16	0,165976753	0,279589565
**-2,22E-16**	-2,22E-16	0,060051315	0,126617751
**-2,22E-16**	0,219354963	**0,153698511**	0,457806913
**0,059313637**	0,175495058	0,22850512	0,34863876
**0,077826985**	0,05712438	**0,165648602**	0,224981314
**0,045701888**	0,108202901	**0,145743002**	**0,277603394**
**0,121934397**	0,196463252	0,190917352	0,364408239
0,084860487	0,123247811	**0,205022683**	0,367337096
**0,050609685**	-2,22E-16	0,495149928	0,024597227
**-2,22E-16**	0,092084263	**-2,22E-16**	0,466706302
**0,022800295**	-2,22E-16	0,161613716	0,264523252
0,219250695	-2,22E-16	0,191589037	-2,22E-16
**0,0519306**	0,094440309	**0,129613719**	0,411398999
**0,134744782**	-2,22E-16	**0,328041074**	0,059587279
**0,081972652**	0,051132473	**0,182294358**	0,342230342
**0,087119536**	0,105857012	**0,167931903**	0,257930023
0,060133438	0,062943616	**0,133361514**	0,309933684
**0,119694117**	**0,122221427**	0,265036416	**0,190944162**
0,109524127	2,22E-16	**0,291866633**	0,073346001
-2,22E-16	0,046292186	0,273190385	0,351893468
**0,090008933**	**0,055931576**	0,275212919	0,229693043
**0,108476429**	**0,033558577**	0,231729806	0,261921976
**0,337458804**	-2,22E-16	**0,532555416**	-2,22E-16
**-2,22E-16**	-2,22E-16	**0,315608942**	**0,071997206**
0,387448856	**-2,22E-16**	0,097695931	0,023767481

Elleanthus_lancifolius	Sobralia_albolutea	Sobralia_antioquiensis	Sobralia_bradeorum
**0,209672507**			
**0,164015094**	**0,202196984**		
0,10938082	0,279991095	**0,132753697**	
**0,336997837**	**0,113651076**	**0,164616697**	**0,299377882**
0,579729738	0,338245761	0,100777377	**0,078522216**
**0,438571438**	0,226163514	0,24997797	**0,203785335**
0,501937643	**0,206933207**	0,153355473	**0,329974512**
**0,500893911**	**0,243952906**	0,174035566	**0,292169027**
0,217562776	0,135540848	0,09247207	0,184523455
**0,694389177**	**0,264713326**	0,067492589	0,080192704
0,678877525	0,20939179	0,204718183	0,275794794
0,611003373	0,303076422	0,185805375	0,26436792
**0,53032011**	**0,213409206**	0,199904749	0,300992293
**0,194222564**	**0,413613902**	0,090580543	**0,187164225**
**0,756823572**	**0,322206023**	0,164392078	**0,138101069**
**0,694707613**	**0,145546481**	**0,114750375**	0,221707457
**0,600899655**	**0,182168376**	0,124325753	**0,267502519**
**0,360104906**	**0,229215069**	0,014426074	**0,226404014**
**0,669520355**	**0,237087658**	0,08549824	**0,270476914**
0,753377049	0,253339431	0,181749328	**0,316948679**
0,217805855	0,103702928	0,027109152	**0,331112806**
0,472081086	0,297764249	0,037928476	**0,090987879**
**0,409511771**	**0,366170121**	0,28138093	**0,248782985**
0,091887713	0,003477335	-2,22E-16	**0,268542785**
0,628655285	0,213199824	0,166173919	**0,208673674**
**0,396637588**	**0,079281735**	**0,184232704**	**0,315362622**
**0,611947715**	**0,321890494**	0,230265527	**0,235299277**
**0,526980468**	**0,128583221**	0,111007813	**0,223415535**
0,659148508	**0,214915344**	0,102814549	**0,223769353**
**0,528653073**	**0,21944539**	**0,149016071**	**0,313713582**
**0,381709494**	0,089105789	0,137793901	**0,316189379**
0,354240448	0,41852939	0,457925929	0,306351927
0,565524366	0,188410913	0,213723711	0,323259131
0,455893951	0,187255325	0,192202493	0,317602655
0,118789054	0,005880991	-2,22E-16	0,575704097
**0,09207699**	**0,103367815**	-2,22E-16	0,074898157
0,122698055	0,072045326	-2,22E-16	**0,332870464**

Sobralia_candida	Sobralia_chrysostoma	Sobralia_corazoi	Sobralia_crocea	Sobralia_decora
**0,141990409**				
0,589602635	**0,173617201**			
0,649385202	**0,351273142**	**0,467682856**		
**0,550364136**	0,279616171	**0,507152535**	**0,543660125**	
0,555411697	0,067998773	0,640213663	0,424797035	**0,571447402**
0,079606633	**0,840833768**	**0,175212663**	**0,341052421**	0,269885257
0,649558561	0,382321845	0,53253511	**0,607244235**	0,803943005
0,550036204	0,422117248	0,567939442	**0,57625863**	0,742697201
0,688852971	0,258177931	**0,482949983**	**0,706528876**	0,44023648
0,197835315	**0,161335614**	0,420158474	**0,248355827**	0,382937433
**0,305323444**	**0,763869353**	**0,321866577**	**0,506285169**	**0,469156864**
0,329063241	0,694071144	0,316283579	**0,418534615**	0,489008709
0,641768927	0,306645891	**0,422796725**	0,72440786	0,679346257
**0,398617186**	**0,468003861**	**0,269810579**	**0,567719492**	**0,448290574**
0,417956221	0,699663506	0,294629714	0,488595456	0,596553589
0,603610516	0,489950975	0,495839284	0,636313623	0,664391766
0,27651662	0,088701996	0,306788112	**0,254821742**	0,364744814
0,11208006	0,669077002	0,199965176	**0,285048766**	0,250068667
0,36434335	**0,380849914**	0,585119568	**0,347921111**	0,588073194
0,358937629	0,0085614	0,168530164	**0,206917925**	0,515795248
0,503314976	0,561068829	0,46649505	**0,581802479**	0,581244349
**0,562612227**	0,23763825	0,554514846	0,51257111	0,72024634
0,568492791	**0,461848467**	0,428211806	0,549975312	0,729440871
0,553983129	**0,399632085**	0,371695303	**0,64134523**	0,529927878
0,603376105	**0,444486827**	0,474807903	**0,672904552**	0,707644622
**0,709145021**	**0,408459501**	**0,465001362**	**0,703165479**	**0,777202603**
**0,727520165**	0,080506803	0,5123607	0,680668212	**0,609166794**
0,357894634	0,247054773	0,574833423	0,386580767	0,436660237
0,64648997	0,341947845	0,595277166	0,644760841	0,807245188
0,667582754	0,290322035	0,505966909	0,479393123	0,811278294
0,388178235	0,014528707	0,183689698	0,324365192	0,365589963
0,116028134	0,071697127	0,184206552	0,125033809	0,159030256
0,32231106	0,037246295	0,293705087	0,23937127	0,406364469

Sobralia_ecuadorana	Sobralia_elisabethae	Sobralia_fenzliana	Sobralia_fragrans	Sobralia_gloriosa
**0,093777509**				
0,492255986	**0,348621064**			
0,530931551	0,496973844	**0,66519769**		
0,313614016	0,192380492	0,601054551	**0,423129833**	
0,529211625	0,241363838	0,216668975	0,353132078	**0,18081503**
**0,186308337**	**0,637604404**	**0,590030385**	**0,601158501**	**0,482733352**
0,301142775	**0,725792833**	0,473538433	0,61103159	0,271998786
**0,43685145**	0,453109598	**0,760759537**	**0,579539767**	**0,541168489**
**0,244137076**	0,419900538	**0,529515137**	**0,352722427**	**0,425488601**
0,339231634	0,667649486	0,61336522	**0,709009173**	0,384612571
0,407801566	0,645760711	0,743040211	0,737950576	0,504543478
0,431867133	0,110164273	0,266154171	0,420492225	0,173724972
0,040936101	0,465678223	0,290867035	0,40644254	0,267554948
**0,516284085**	0,317617118	0,560226666	**0,68166859**	**0,258337122**
**0,339159295**	0,037261649	0,505931732	0,482757114	0,081283042
**0,315832971**	0,545423833	0,698675325	**0,593637113**	0,481259796
**0,467432269**	**0,189157524**	**0,616728117**	**0,657881991**	**0,349046158**
0,442064322	0,370517469	0,705436846	**0,805359204**	**0,537566048**
**0,424767938**	0,406116704	0,730817163	**0,567733037**	**0,495667368**
**0,399147594**	0,578508711	0,774607523	**0,667461513**	**0,55956184**
**0,504700694**	**0,210953473**	**0,731435031**	**0,564441207**	**0,61460402**
**0,398232408**	**0,061270892**	0,658709655	0,38204121	**0,538783226**
0,308534442	0,252582913	0,398056005	0,466474566	0,482346784
0,565647073	0,371888103	0,736897063	0,695962414	0,618156644
0,459189317	0,224322409	0,8287801	0,610313445	0,6534645
0,218802234	0,055162215	0,308562053	0,229804706	0,254799038
0,168405114	0,083640704	0,119556293	0,172503159	0,122360745
0,308978722	0,088135413	0,260093716	0,311906034	0,122159252

Sobralia_granitica	Sobralia_helleri	Sobralia_infundibuligera	Sobralia_klotscheana	Sobralia_lancea
**0,227614905**				
**0,265584667**	**0,43087396**			
**0,162665166**	0,515955954	**0,536621285**		
**0,046559867**	**0,478154188**	**0,370783339**	**0,427347946**	
**0,215342624**	0,783667745	**0,688687408**	**0,558358569**	**0,46376522**
0,265305805	**0,650152353**	**0,630823386**	0,637334656	0,517482443
**0,305240502**	0,193201177	0,219264037	**0,23322038**	0,086984669
**0,083398608**	**0,675029055**	**0,341013213**	**0,368634937**	0,514808016
**0,546561211**	**0,636520846**	**0,293467497**	**0,40607303**	0,230376377
**0,103378013**	**0,082848169**	**0,227768411**	**0,411462676**	**0,054428629**
**0,202891926**	**0,58995547**	**0,631055649**	0,725835247	**0,454380529**
**0,246953116**	**0,27205998**	0,467902591	**0,573025297**	**0,344444072**
**0,280038934**	0,672495401	0,517828947	**0,632077813**	**0,433380134**
**0,175707568**	0,427040978	**0,494716635**	**0,653812003**	0,415440888
**0,209002392**	**0,613235473**	**0,655268515**	0,646016158	**0,481231261**
**0,246628332**	**0,47499273**	**0,489892217**	**0,772776787**	**0,574766684**
0,171324308	**0,234818299**	0,326663735	0,596077225	0,343185787
0,485047998	0,480051223	0,230939121	0,309895348	0,115043418
0,325010614	0,51188351	0,505843694	0,744638752	0,433945971
0,244665191	0,501688794	0,425157907	0,758254895	0,425432826
0,062275498	0,105146571	0,177341946	0,232064225	0,209817595
0,200713726	0,09561078	0,097167275	0,085840459	-2,22E-16
0,432001851	0,092823088	0,261132732	0,244265277	0,053119514

Sobralia_leucoxantha	Sobralia_liliastrum	Sobralia_lindleyana	Sobralia_luerorum	Sobralia_luteola
**0,708554749**				
0,300104169	**0,261888455**			
**0,511125109**	0,344525256	**0,076717277**		
0,488459323	0,546031638	0,483682025	**0,314413851**	
0,290912107	0,278409627	0,36722342	-2,22E-16	**0,202940443**
0,614721902	0,789747968	0,201443161	0,322288282	0,606933258
**0,37168529**	0,476317862	**0,361742441**	**0,045621287**	0,325944772
0,655835698	0,675911096	0,320052299	0,410342882	0,610962691
0,481192298	0,559149389	0,205734671	0,355773782	0,339198713
0,561875936	0,722712496	0,199472555	**0,295923163**	0,475207937
**0,57996072**	**0,692129104**	**0,297076803**	**0,271520001**	**0,511705369**
0,308232537	**0,468421298**	**0,233023213**	**0,058562155**	0,324209926
0,323902762	0,448926775	0,369955579	0,295225261	0,669546399
0,557506196	0,745946316	0,321419878	0,342355203	0,521203786
0,448939727	0,659278165	0,276254064	0,385042875	0,473389371
0,282472658	0,271649611	0,349704025	-2,22E-16	0,139392028
0,088720178	0,110995307	0,287030622	-2,22E-16	0,196744601
0,232385884	0,251248392	0,382304433	-2,22E-16	0,285984786

Sobralia_macrantha	Sobralia_macrophylla	Sobralia_mucronata	Sobralia_powellii
**0,281998105**			
**0,511801332**	**0,533010573**		
0,31417513	0,77351982	**0,547174396**	
0,337893498	**0,544348457**	0,559911931	**0,591091847**
0,313382676	**0,76556318**	0,515040596	0,680385196
**0,353273837**	**0,630298978**	**0,658628678**	**0,647342874**
0,375689153	**0,4310805**	0,643288721	0,503735222
0,107436235	0,369406071	0,298218052	0,454960819
0,430372406	0,76692548	0,726227723	0,756104087
0,455240661	0,50010603	0,502274251	0,670229231
0,426792791	0,1405024	0,295291345	0,182964997
0,092703101	0,097196169	0,154030048	0,115654684
0,416562848	0,149515126	0,297983473	0,261368409

Sobralia_pulcherrima	Sobralia_rosea	Sobralia_sessilis	Sobralia_setigera	Sobralia_suaveolens
**0,621286272**				
**0,621025191**	**0,659392841**			
0,554288104	**0,619963896**	**0,678849971**		
0,25044709	0,327471715	0,38327908	**0,319196369**	
0,478426064	0,783100835	0,761696142	**0,671250752**	**0,42049307**
0,662386779	0,597354654	0,778287603	0,746198636	**0,4496043**
0,236792894	0,187057972	0,376628396	0,417849411	**0,148386755**
0,097193817	0,075189336	0,175157998	**0,099591811**	**0,298573291**
0,199240946	0,202598565	0,325088649	**0,288308979**	**0,216215666**

Sobralia_valida	Sobralia_violacea	Sobralia_warszewiczii	Sobralia_wilsoniana
**0,790489027**			
0,360769868	**0,329558605**		
**0,1530884**	**0,103862658**	**0,18799558**	
**0,290566662**	0,256191718	0,387618492	**0,169205877**

X	Brasolia_cattleya	Brasolia_ciliata	Brasolia_dichotoma	Brasolia_dorbignyana
Brasolia_ciliata	**0,63334183**			
Brasolia_dichotoma	**0,432456462**	**0,489693626**		
Brasolia_dorbignyana	0,454223228	**0,580664373**	**0,604367741**	
Brasolia_rupicola	0,090672087	**0,037369319**	0,201346073	**0,16651347**
Brasolia_speciosa	**2,22E-16**	**-2,22E-16**	**-2,22E-16**	**-2,22E-16**
Brasolia_stenophylla	**0,28340298**	**0,125679843**	**0,04840082**	0,225564741
Elleanthus_ampliflorus	0,098935615	-2,22E-16	0,002720348	**0,0141526**
Elleanthus_lancifolius	0,427037407	0,239750756	0,070177913	0,161912545
Sobralia_albolutea	**0,057396627**	**-2,22E-16**	**0,010791013**	**-2,22E-16**
Sobralia_antioquiensis	**0,187799677**	**-2,22E-16**	**0,024887272**	**-2,22E-16**
Sobralia_bradeorum	**0,307079186**	**0,272824102**	**0,384156175**	**0,326748586**
Sobralia_candida	0,634276491	0,442400976	**0,502016048**	**0,557628879**
Sobralia_chrysostoma	0,04231589	-2,22E-16	**0,014394553**	**0,010923616**
Sobralia_corazoi	**0,503726931**	0,426880669	0,216810698	**0,342622349**
Sobralia_crocea	0,633550697	0,534137107	**0,428241209**	**0,488068411**
Sobralia_decora	**0,427981285**	**0,317484601**	0,296196723	**0,397592862**
Sobralia_ecuadorana	0,468157563	0,343442078	0,296251323	**0,389637681**
Sobralia_elisabethae	0,061495231	0,007242533	**0,0190861**	**0,021613649**
Sobralia_fenzliana	0,563456367	0,455953899	**0,386138872**	**0,512601217**
Sobralia_fragrans	**0,371716988**	0,161079805	0,076268093	**0,18627737**
Sobralia_gloriosa	0,534057533	0,49938075	0,449372297	**0,445263615**
Sobralia_granitica	0,057337805	-2,22E-16	0,020792421	**0,002798512**
Sobralia_helleri	**0,20720666**	**0,083113307**	**0,027735978**	**0,064599928**
Sobralia_infundibuligera	**0,328216257**	**0,247028072**	0,101032677	**0,1971685**
Sobralia_klotscheana	**0,509156899**	**0,378352013**	0,371061843	**0,390589276**
Sobralia_lancea	**0,393512511**	**0,569460331**	**0,395947507**	**0,38576134**
Sobralia_leucoxantha	0,225133942	0,160063152	**0,1500813**	**0,22329764**
Sobralia_liliastrum	**0,390298671**	0,251957514	**0,261922281**	**0,274414369**
Sobralia_lindleyana	0,152930477	0,027389015	**0,023793292**	**0,158353691**
Sobralia_luerorum	**0,047246819**	0,034441992	**0,006202832**	**0,022565062**
Sobralia_luteola	0,203616747	0,017155457	**0,041101163**	**0,050681563**
Sobralia_macrantha	**0,121487141**	**0,067037065**	**0,299200868**	**0,379149381**
Sobralia_macrophylla	**0,377000915**	**0,275545949**	**0,116828453**	**0,198777281**
Sobralia_mucronata	0,492010458	**0,421440368**	0,217582622	0,347571219
Sobralia_powellii	0,379529181	0,199255636	0,109309752	0,202166839
Sobralia_pulcherrima	**0,460994717**	**0,206799818**	**0,305654118**	**0,475225567**
Sobralia_rosea	**0,470819613**	**0,342769073**	**0,278233423**	**0,435182783**
Sobralia_sessilis	**0,608053868**	**0,462372329**	0,468779471	**0,615244295**
Sobralia_setigera	**0,678517923**	**0,529594604**	**0,565939857**	**0,70263688**
Sobralia_suaveolens	**0,283410386**	**0,034209387**	0,057599964	0,054337446
Sobralia_valida	**0,609708066**	**0,514985334**	**0,328063348**	**0,421216675**
Sobralia_violacea	0,579849826	**0,395494632**	**0,424105805**	0,61762324
Sobralia_warszewiczii	0,507078055	**0,300239727**	0,544058017	**0,474058731**
Sobralia_wilsoniana	**0,124282942**	**-2,22E-16**	**0,054296119**	**0,052062288**
Sobralia_xantholeuca	**0,124715008**	**0,062562197**	**0,262755656**	0,189309893

Brasolia_rupicola	Brasolia_speciosa	Brasolia_stenophylla	Elleanthus_ampliflorus
**-2,22E-16**			
-2,22E-16	**-2,22E-16**		
**-2,22E-16**	0,180830634	**0,009532849**	
**0,001941015**	0,137700165	0,14797935	**0,400701962**
**-2,22E-16**	2,22E-16	**0,018942935**	0,181092118
**-2,22E-16**	-2,22E-16	**2,22E-16**	0,212990611
**0,365935456**	-2,22E-16	**0,228205859**	0,141077648
**0,097912061**	**-2,22E-16**	0,225330106	**0,100942248**
**-2,22E-16**	0,267247917	0,02374286	0,253947088
**2,22E-16**	2,22E-16	0,26474368	0,156615839
**0,086402584**	0,098849748	**0,251552792**	0,339225487
**0,108726966**	0,061085079	0,241726103	0,21048403
**0,004678628**	-2,22E-16	0,273226981	0,076173139
**-2,22E-16**	0,199762772	0,104715759	0,410573735
**0,108080998**	0,086002464	0,184105241	0,291219236
0,066720607	0,067022413	0,270880278	0,270999673
**0,019039751**	-2,22E-16	0,165976753	0,279589565
**-2,22E-16**	-2,22E-16	0,060051315	0,126617751
**-2,22E-16**	0,219354963	**0,153698511**	0,457806913
**0,059313637**	0,175495058	0,22850512	0,34863876
**0,077826985**	0,05712438	**0,165648602**	0,224981314
**0,045701888**	0,108202901	**0,145743002**	**0,277603394**
**0,121934397**	0,196463252	0,190917352	0,364408239
0,084860487	0,123247811	**0,205022683**	0,367337096
**0,050609685**	-2,22E-16	0,495149928	0,024597227
**-2,22E-16**	0,092084263	**-2,22E-16**	0,466706302
**0,022800295**	-2,22E-16	0,161613716	0,264523252
0,219250695	-2,22E-16	0,191589037	-2,22E-16
**0,0519306**	0,094440309	**0,129613719**	0,411398999
**0,134744782**	-2,22E-16	**0,328041074**	0,059587279
**0,081972652**	0,051132473	**0,182294358**	0,342230342
**0,087119536**	0,105857012	**0,167931903**	0,257930023
0,060133438	0,062943616	**0,133361514**	0,309933684
**0,119694117**	**0,122221427**	0,265036416	**0,190944162**
0,109524127	2,22E-16	**0,291866633**	0,073346001
-2,22E-16	0,046292186	0,273190385	0,351893468
**0,090008933**	**0,055931576**	0,275212919	0,229693043
**0,108476429**	**0,033558577**	0,231729806	0,261921976
**0,337458804**	-2,22E-16	**0,532555416**	-2,22E-16
**-2,22E-16**	-2,22E-16	**0,315608942**	**0,071997206**
0,387448856	**-2,22E-16**	0,097695931	0,023767481

Elleanthus_lancifolius	Sobralia_albolutea	Sobralia_antioquiensis	Sobralia_bradeorum
**0,209672507**			
**0,164015094**	**0,202196984**		
0,10938082	0,279991095	**0,132753697**	
**0,336997837**	**0,113651076**	**0,164616697**	**0,299377882**
0,579729738	0,338245761	0,100777377	**0,078522216**
**0,438571438**	0,226163514	0,24997797	**0,203785335**
0,501937643	**0,206933207**	0,153355473	**0,329974512**
**0,500893911**	**0,243952906**	0,174035566	**0,292169027**
0,217562776	0,135540848	0,09247207	0,184523455
**0,694389177**	**0,264713326**	0,067492589	0,080192704
0,678877525	0,20939179	0,204718183	0,275794794
0,611003373	0,303076422	0,185805375	0,26436792
**0,53032011**	**0,213409206**	0,199904749	0,300992293
**0,194222564**	**0,413613902**	0,090580543	**0,187164225**
**0,756823572**	**0,322206023**	0,164392078	**0,138101069**
**0,694707613**	**0,145546481**	**0,114750375**	0,221707457
**0,600899655**	**0,182168376**	0,124325753	**0,267502519**
**0,360104906**	**0,229215069**	0,014426074	**0,226404014**
**0,669520355**	**0,237087658**	0,08549824	**0,270476914**
0,753377049	0,253339431	0,181749328	**0,316948679**
0,217805855	0,103702928	0,027109152	**0,331112806**
0,472081086	0,297764249	0,037928476	**0,090987879**
**0,409511771**	**0,366170121**	0,28138093	**0,248782985**
0,091887713	0,003477335	-2,22E-16	**0,268542785**
0,628655285	0,213199824	0,166173919	**0,208673674**
**0,396637588**	**0,079281735**	**0,184232704**	**0,315362622**
**0,611947715**	**0,321890494**	0,230265527	**0,235299277**
**0,526980468**	**0,128583221**	0,111007813	**0,223415535**
0,659148508	**0,214915344**	0,102814549	**0,223769353**
**0,528653073**	**0,21944539**	**0,149016071**	**0,313713582**
**0,381709494**	0,089105789	0,137793901	**0,316189379**
0,354240448	0,41852939	0,457925929	0,306351927
0,565524366	0,188410913	0,213723711	0,323259131
0,455893951	0,187255325	0,192202493	0,317602655
0,118789054	0,005880991	-2,22E-16	0,575704097
**0,09207699**	**0,103367815**	-2,22E-16	0,074898157
0,122698055	0,072045326	-2,22E-16	**0,332870464**

Sobralia_candida	Sobralia_chrysostoma	Sobralia_corazoi	Sobralia_crocea	Sobralia_decora
**0,141990409**				
0,589602635	**0,173617201**			
0,649385202	**0,351273142**	**0,467682856**		
**0,550364136**	0,279616171	**0,507152535**	**0,543660125**	
0,555411697	0,067998773	0,640213663	0,424797035	**0,571447402**
0,079606633	**0,840833768**	**0,175212663**	**0,341052421**	0,269885257
0,649558561	0,382321845	0,53253511	**0,607244235**	0,803943005
0,550036204	0,422117248	0,567939442	**0,57625863**	0,742697201
0,688852971	0,258177931	**0,482949983**	**0,706528876**	0,44023648
0,197835315	**0,161335614**	0,420158474	**0,248355827**	0,382937433
**0,305323444**	**0,763869353**	**0,321866577**	**0,506285169**	**0,469156864**
0,329063241	0,694071144	0,316283579	**0,418534615**	0,489008709
0,641768927	0,306645891	**0,422796725**	0,72440786	0,679346257
**0,398617186**	**0,468003861**	**0,269810579**	**0,567719492**	**0,448290574**
0,417956221	0,699663506	0,294629714	0,488595456	0,596553589
0,603610516	0,489950975	0,495839284	0,636313623	0,664391766
0,27651662	0,088701996	0,306788112	**0,254821742**	0,364744814
0,11208006	0,669077002	0,199965176	**0,285048766**	0,250068667
0,36434335	**0,380849914**	0,585119568	**0,347921111**	0,588073194
0,358937629	0,0085614	0,168530164	**0,206917925**	0,515795248
0,503314976	0,561068829	0,46649505	**0,581802479**	0,581244349
**0,562612227**	0,23763825	0,554514846	0,51257111	0,72024634
0,568492791	**0,461848467**	0,428211806	0,549975312	0,729440871
0,553983129	**0,399632085**	0,371695303	**0,64134523**	0,529927878
0,603376105	**0,444486827**	0,474807903	**0,672904552**	0,707644622
**0,709145021**	**0,408459501**	**0,465001362**	**0,703165479**	**0,777202603**
**0,727520165**	0,080506803	0,5123607	0,680668212	**0,609166794**
0,357894634	0,247054773	0,574833423	0,386580767	0,436660237
0,64648997	0,341947845	0,595277166	0,644760841	0,807245188
0,667582754	0,290322035	0,505966909	0,479393123	0,811278294
0,388178235	0,014528707	0,183689698	0,324365192	0,365589963
0,116028134	0,071697127	0,184206552	0,125033809	0,159030256
0,32231106	0,037246295	0,293705087	0,23937127	0,406364469

Sobralia_ecuadorana	Sobralia_elisabethae	Sobralia_fenzliana	Sobralia_fragrans	Sobralia_gloriosa
**0,093777509**				
0,492255986	**0,348621064**			
0,530931551	0,496973844	**0,66519769**		
0,313614016	0,192380492	0,601054551	**0,423129833**	
0,529211625	0,241363838	0,216668975	0,353132078	**0,18081503**
**0,186308337**	**0,637604404**	**0,590030385**	**0,601158501**	**0,482733352**
0,301142775	**0,725792833**	0,473538433	0,61103159	0,271998786
**0,43685145**	0,453109598	**0,760759537**	**0,579539767**	**0,541168489**
**0,244137076**	0,419900538	**0,529515137**	**0,352722427**	**0,425488601**
0,339231634	0,667649486	0,61336522	**0,709009173**	0,384612571
0,407801566	0,645760711	0,743040211	0,737950576	0,504543478
0,431867133	0,110164273	0,266154171	0,420492225	0,173724972
0,040936101	0,465678223	0,290867035	0,40644254	0,267554948
**0,516284085**	0,317617118	0,560226666	**0,68166859**	**0,258337122**
**0,339159295**	0,037261649	0,505931732	0,482757114	0,081283042
**0,315832971**	0,545423833	0,698675325	**0,593637113**	0,481259796
**0,467432269**	**0,189157524**	**0,616728117**	**0,657881991**	**0,349046158**
0,442064322	0,370517469	0,705436846	**0,805359204**	**0,537566048**
**0,424767938**	0,406116704	0,730817163	**0,567733037**	**0,495667368**
**0,399147594**	0,578508711	0,774607523	**0,667461513**	**0,55956184**
**0,504700694**	**0,210953473**	**0,731435031**	**0,564441207**	**0,61460402**
**0,398232408**	**0,061270892**	0,658709655	0,38204121	**0,538783226**
0,308534442	0,252582913	0,398056005	0,466474566	0,482346784
0,565647073	0,371888103	0,736897063	0,695962414	0,618156644
0,459189317	0,224322409	0,8287801	0,610313445	0,6534645
0,218802234	0,055162215	0,308562053	0,229804706	0,254799038
0,168405114	0,083640704	0,119556293	0,172503159	0,122360745
0,308978722	0,088135413	0,260093716	0,311906034	0,122159252

Sobralia_granitica	Sobralia_helleri	Sobralia_infundibuligera	Sobralia_klotscheana	Sobralia_lancea
**0,227614905**				
**0,265584667**	**0,43087396**			
**0,162665166**	0,515955954	**0,536621285**		
**0,046559867**	**0,478154188**	**0,370783339**	**0,427347946**	
**0,215342624**	0,783667745	**0,688687408**	**0,558358569**	**0,46376522**
0,265305805	**0,650152353**	**0,630823386**	0,637334656	0,517482443
**0,305240502**	0,193201177	0,219264037	**0,23322038**	0,086984669
**0,083398608**	**0,675029055**	**0,341013213**	**0,368634937**	0,514808016
**0,546561211**	**0,636520846**	**0,293467497**	**0,40607303**	0,230376377
**0,103378013**	**0,082848169**	**0,227768411**	**0,411462676**	**0,054428629**
**0,202891926**	**0,58995547**	**0,631055649**	0,725835247	**0,454380529**
**0,246953116**	**0,27205998**	0,467902591	**0,573025297**	**0,344444072**
**0,280038934**	0,672495401	0,517828947	**0,632077813**	**0,433380134**
**0,175707568**	0,427040978	**0,494716635**	**0,653812003**	0,415440888
**0,209002392**	**0,613235473**	**0,655268515**	0,646016158	**0,481231261**
**0,246628332**	**0,47499273**	**0,489892217**	**0,772776787**	**0,574766684**
0,171324308	**0,234818299**	0,326663735	0,596077225	0,343185787
0,485047998	0,480051223	0,230939121	0,309895348	0,115043418
0,325010614	0,51188351	0,505843694	0,744638752	0,433945971
0,244665191	0,501688794	0,425157907	0,758254895	0,425432826
0,062275498	0,105146571	0,177341946	0,232064225	0,209817595
0,200713726	0,09561078	0,097167275	0,085840459	-2,22E-16
0,432001851	0,092823088	0,261132732	0,244265277	0,053119514

Sobralia_leucoxantha	Sobralia_liliastrum	Sobralia_lindleyana	Sobralia_luerorum	Sobralia_luteola
**0,708554749**				
0,300104169	**0,261888455**			
**0,511125109**	0,344525256	**0,076717277**		
0,488459323	0,546031638	0,483682025	**0,314413851**	
0,290912107	0,278409627	0,36722342	-2,22E-16	**0,202940443**
0,614721902	0,789747968	0,201443161	0,322288282	0,606933258
**0,37168529**	0,476317862	**0,361742441**	**0,045621287**	0,325944772
0,655835698	0,675911096	0,320052299	0,410342882	0,610962691
0,481192298	0,559149389	0,205734671	0,355773782	0,339198713
0,561875936	0,722712496	0,199472555	**0,295923163**	0,475207937
**0,57996072**	**0,692129104**	**0,297076803**	**0,271520001**	**0,511705369**
0,308232537	**0,468421298**	**0,233023213**	**0,058562155**	0,324209926
0,323902762	0,448926775	0,369955579	0,295225261	0,669546399
0,557506196	0,745946316	0,321419878	0,342355203	0,521203786
0,448939727	0,659278165	0,276254064	0,385042875	0,473389371
0,282472658	0,271649611	0,349704025	-2,22E-16	0,139392028
0,088720178	0,110995307	0,287030622	-2,22E-16	0,196744601
0,232385884	0,251248392	0,382304433	-2,22E-16	0,285984786

Sobralia_macrantha	Sobralia_macrophylla	Sobralia_mucronata	Sobralia_powellii
**0,281998105**			
**0,511801332**	**0,533010573**		
0,31417513	0,77351982	**0,547174396**	
0,337893498	**0,544348457**	0,559911931	**0,591091847**
0,313382676	**0,76556318**	0,515040596	0,680385196
**0,353273837**	**0,630298978**	**0,658628678**	**0,647342874**
0,375689153	**0,4310805**	0,643288721	0,503735222
0,107436235	0,369406071	0,298218052	0,454960819
0,430372406	0,76692548	0,726227723	0,756104087
0,455240661	0,50010603	0,502274251	0,670229231
0,426792791	0,1405024	0,295291345	0,182964997
0,092703101	0,097196169	0,154030048	0,115654684
0,416562848	0,149515126	0,297983473	0,261368409

Sobralia_pulcherrima	Sobralia_rosea	Sobralia_sessilis	Sobralia_setigera	Sobralia_suaveolens
**0,621286272**				
**0,621025191**	**0,659392841**			
0,554288104	**0,619963896**	**0,678849971**		
0,25044709	0,327471715	0,38327908	**0,319196369**	
0,478426064	0,783100835	0,761696142	**0,671250752**	**0,42049307**
0,662386779	0,597354654	0,778287603	0,746198636	**0,4496043**
0,236792894	0,187057972	0,376628396	0,417849411	**0,148386755**
0,097193817	0,075189336	0,175157998	**0,099591811**	**0,298573291**
0,199240946	0,202598565	0,325088649	**0,288308979**	**0,216215666**

Sobralia_valida	Sobralia_violacea	Sobralia_warszewiczii	Sobralia_wilsoniana
**0,790489027**			
0,360769868	**0,329558605**		
**0,1530884**	**0,103862658**	**0,18799558**	
**0,290566662**	0,256191718	0,387618492	**0,169205877**
