# Supplementary material for: Niche conservatism and evolution of climatic tolerance in the Neotropical orchid genera Sobralia and Brasolia (Orchidaceae)
Source: Sci Rep. 2022 Aug 17;12:13936. doi: 10.1038/s41598-022-18218-4 (PMC9385687; doi:10.1038/s41598-022-18218-4)
Supplement: Supplementary file 8 — Supplementary Information 8. [file 41598_2022_18218_MOESM8_ESM.docx]

**Annex 1.** Current distribution of suitable habitats of *Brasolia* representatives: *B. cattleya* (A), *B. ciliata* (B), *B. dichotoma* (C), *B. flava* (D), *B. rupicola* (E), *B. speciosa* (F), and *B. stenophylla* (G).

**Annex 2.** Current distribution of suitable habitats of *Sobralia sect. Intermediae and unclassified S. valida* representatives: *S. corazoi* (A), *S. crocea* (B), *S. fragrans* (C) *S. lancea* (D), *S. mucronata* (E), *S. suaveolens* (F), and *S. valida* (G).

**Annex 3.** Current distribution of suitable habitats of *Sobralia sect.* Racemosae representatives and unclassified S. luteola representatives: *S. elisabethae* (A), *S. gloriosa* (B), *S. granitica* (C), *S. liliastrum* (D), *S. pulcherrima* (E), *S. rosea* (F) and *S. luteola* (E).

**Annex 4.** Current distribution of suitable habitats of *Sobralia sect. Sobralia 1* representatives: *S. albolutea* (A), *S. decora* (B), *S. fenzliana* (C), *S. klotscheana* (D), *S. sessilis* (E), *S. violacea* (F), and *S. warszewiczii* (G).

**Annex 5.** Current distribution of suitable habitats of *Sobralia sect. Sobralia 2* representatives: *S. bradeorum* (A), *S. chrysostoma* (B), *S. ecuadorana* (C), *S. helleri* (D), *S. leucoxantha* (E), *S. powellii* (F), and *S. setigera* (G).

**Annex 6.** Current distribution of suitable habitats of *Sobralia sect. Sobralia 3* representatives and unclassified *S. infundibuligera*, *S. lindleyana* and *S. macrophylla*: *S. antioquiensis* (A), *S. macrantha* (B), *S. xantholeuca* (C), *S. wilsoniana* (D), *S. infundibuligera* (E), *S. lindleyana* (F), *and S. macrophylla* (G)
